# Genome-wide identification of microRNAs in pomegranate (*Punica granatum* L.) by high-throughput sequencing

**DOI:** 10.1186/s12870-016-0807-3

**Published:** 2016-05-26

**Authors:** Thangasamy Saminathan, Abiodun Bodunrin, Nripendra V. Singh, Ramajayam Devarajan, Padma Nimmakayala, Moersfelder Jeff, Mallikarjuna Aradhya, Umesh K. Reddy

**Affiliations:** Department of Biology, Gus R. Douglass Institute, West Virginia State University, Institute, WV 25112-1000 USA; ICAR-National Research Center on Pomegranate, Kegaon, Solapur, Maharashtra 413255 India; ICAR-Indian Institute of Oil Palm Research, Pedavegi, West Godavari, Andhra Pradesh 534450 India; National Clonal Germplasm Repository, USDA-ARS, University of California, Davis, CA 95616 USA

**Keywords:** Pomegranate, MicroRNA, Stem-loop RT-qPCR, Fruit development, High-throughput sequencing

## Abstract

**Background:**

MicroRNAs (miRNAs), a class of small non-coding endogenous RNAs that regulate gene expression post-transcriptionally, play multiple key roles in plant growth and development and in biotic and abiotic stress response. Knowledge and roles of miRNAs in pomegranate fruit development have not been explored.

**Results:**

Pomegranate, which accumulates a large amount of anthocyanins in skin and arils, is valuable to human health, mainly because of its antioxidant properties. In this study, we developed a small RNA library from pooled RNA samples from young seedlings to mature fruits and identified both conserved and pomegranate-specific miRNA from 29,948,480 high-quality reads. For the pool of 15- to 30-nt small RNAs, ~50 % were 24 nt. The miR157 family was the most abundant, followed by miR156, miR166, and miR168, with variants within each family. The base bias at the first position from the 5’ end had a strong preference for U for most 18- to 26-nt sRNAs but a preference for A for 18-nt sRNAs. In addition, for all 24-nt sRNAs, the nucleotide U was preferred (97 %) in the first position. Stem-loop RT-qPCR was used to validate the expression of the predominant miRNAs and novel miRNAs in leaves, male and female flowers, and multiple fruit developmental stages; miR156, miR156a, miR159a, miR159b, and miR319b were upregulated during the later stages of fruit development. Higher expression of miR156 in later fruit developmental may positively regulate anthocyanin biosynthesis by reducing SPL transcription factor. Novel miRNAs showed variation in expression among different tissues. These novel miRNAs targeted different transcription factors and hormone related regulators. Gene ontology and KEGG pathway analyses revealed predominant metabolic processes and catalytic activities, important for fruit development. In addition, KEGG pathway analyses revealed the involvement of miRNAs in ascorbate and linolenic acid, starch and sucrose metabolism; RNA transport; plant hormone signaling pathways; and circadian clock.

**Conclusion:**

Our first and preliminary report of miRNAs will provide information on the synthesis of biochemical compounds of pomegranate for future research. The functions of the targets of the novel miRNAs need further investigation.

**Electronic supplementary material:**

The online version of this article (doi:10.1186/s12870-016-0807-3) contains supplementary material, which is available to authorized users.

## Background

Pomegranate (*Punica granatum* L.), one of the two species within the genus *Punica*, producing a non-climacteric fruit with a low respiration rate [1], is a tropical and subtropical attractive deciduous shrub. Pomegranate was previously placed within its own family Punicaceae, but recent phylogenetic studies have shown that it belongs to Lythraceae. It is one of the oldest edible fruits and has grown naturally from Iran to the Himalayas in northern India since ancient times, although it is native to Iran [2–4]. Although pomegranate is widely cultivated, the five major producers are India, Iran, China, the United States and Turkey [5].

The plant is tolerant of various soil conditions and grows well under sunlight and mild winters [6]. The fruit is a round or spherical in shape, with a fleshy, tubular calyx and leathery skin often deep pink or rich red in color [7]. The inside of the fruit is separated by membranous walls into compartments packed with sac-like structures filled with fleshy juicy, red, pink or whitish pulp called arils, and each aril sac contains one white or red, angular, soft or hard seed [6, 7].

In recent years, pomegranate has become popular for its medicinal properties and its nutritional benefit in the human diet. Pomegranate is a nutrient-dense food source rich in phytochemical compounds. It contains high levels of flavonoids and polyphenols, potent antioxidants offering protection against heart disease and cancer. Because of the health-promoting traits from both the edible and nonedible parts of the fruit in treating a wide range of human diseases such as cancer, diabetes, obesity, Alzheimer disease, and hypertension, pomegranate is considered an important commercial and valuable fruit crop across the world [8, 9]. Metabolome analysis revealed that parts of pomegranate including the fruit peel, juice, root and bark, flowers, leaves and seed contain almost 40 biochemical compounds that are beneficial in different therapies [10]. The compounds include gallotannins, ellagic acid, flavonoids, antioxidants, terpenoids and alkaloids [11–13].

The color of the pomegranate fruit including arils develops from the presence of anthocyanins, water-soluble flavonoid pigments, mostly orange to red and purple/blue [14]. In addition to playing significant roles in plant defense mechanisms [15], anthocyanins are considered valuable to human health because of high antioxidant activity [16], and fruit arils, the edible part of pomegranate, contain the highest quantity of anthocyanins [17]. The biochemical pathway of anthocyanin production has been well documented in numerous plant species, with the involvement of chalcone synthase, chalcone isomerase, and leucoanthocyanidin [18].

In Arabidopsis, the anthocyanin pathway is regulated at the transcription level by transcriptional regulators such as the R2R3-MYB domain, WD40 repeat, and a basic helix-loop-helix (bHLH) [19–21]. The WD40-repeat gene is a functional homologue of Arabidopsis *TTG1* and is involved in regulating anthocyanin biosynthesis during pomegranate fruit development [22]. Recently, anthocyanin biosynthetic genes in red and white pomegranate were cloned and characterized [23] and the expression of key regulatory genes of anthocyanin biosynthesis in pomegranate was analyzed [24].

Plants have two major classes of small regulatory non-coding RNAs. They are small interfering RNAs (siRNAs) and microRNAs (miRNAs), both generated from double-stranded RNA precursors into 20- to 24-nt molecules with the help of Dicers or Dicer-like (DCL) [25]. Many basic aspects of plant development and stresses are controlled by miRNA families [26]. Most of the miRNAs are coded by genes spanning 100–400 nt and further processed by the RNA-induced silencing complex containing Argonaute (AGO) proteins. At the end of processing, depending on the presence of the type of AGO effector protein, the targets can be degraded at the mRNA level or inhibited at the translation level [27]. Bioinformatics analyses revealed at least 21 conserved miRNA families, including miR156, miR159, and miR160, in angiosperms. Plants contain more non-conserved than conserved miRNAs [28], and high-throughput sequencing led to the discovery of non-conserved miRNAs from divergent plant species such as cucurbits, grape, barley and apple [29–34]. miRNAs play key roles in different crops for development and stress response, regulation of anthocyanin accumulation in tomato [35], mediation of nitrogen starvation adaptation in *Arabidopsis thaliana* [36], and elongation of fiber in cotton [37].

Although pomegranate is an important fruit crop with many medicinal properties, the information on miRNAs in pomegranate is lacking. In this study, we report the profiling of miRNAs from seedling to fruit with use of Illumina HiSeq 2000 RNA sequencing and expression analysis of specific miRNAs in leaves, flowers and during fruit development. miR157 was the most abundant miRNA, followed by miR156, miR166, and others. Among different small RNAs (sRNAs), those of 24 nt were most abundant. We found 28 novel miRNAs along with predicted precursor structures and participating pathways. The results from this study could provide valuable information to further reveal the regulatory roles in pomegranate.

## Methods

### Plant materials

**Fig. 1:**
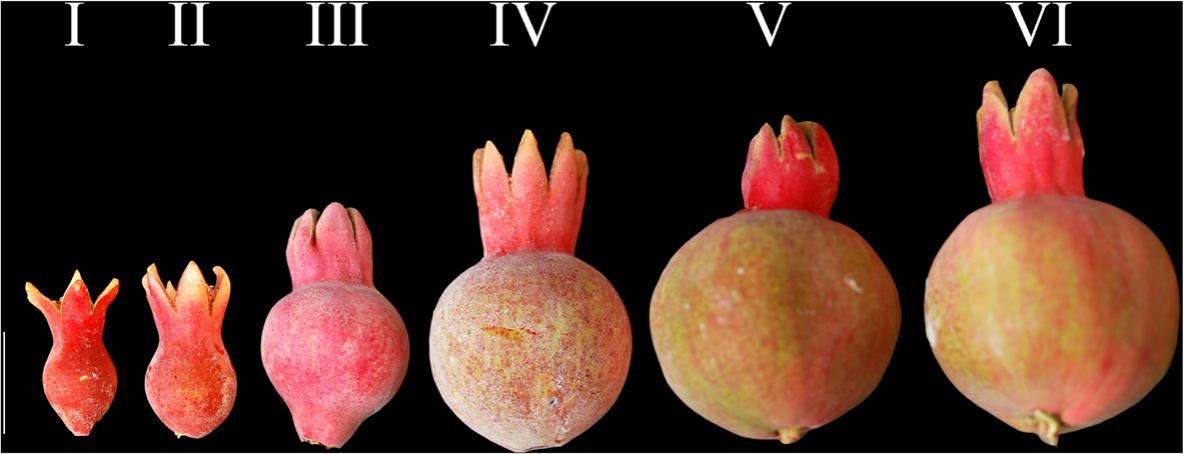
Morphological features of pomegranate fruit development stages. Harvested fruit at different developmental stages from days after pollination divided into six stages. Scale bar: 2 cm

Young leaves, male and female flowers and arils of developing fruits (developmental stages I to VI described in Fig. 1) were collected in 2014 from the cultivar ‘Al-sirin-nar’ grown in the USDA pomegranate germplasm collection at the Wolfskill experimental orchard in Winters, CA, USA (38°50’34.48“ N; 121°97’83.02” W), were immediately frozen in liquid nitrogen, and were finally stored at − 70 °C. For each tissue type, we have collected leaves, flowers, and fruits of different stages from three independent trees. And these three independent trees were considered as biological replications for stem-loop RT-qPCR experiments.

### Collection of arils from mature fruits to grow seedlings

Arils of physiologically mature ‘Al-sirin-nar’ fruits were removed by gently opening the fruits and extracting the arils with the help of air and water. The extracted pomegranate arils were immersed in a bath of cold water, and all other elements of the fruit were washed away. All extracted arils were separated from all other fruit parts, leaving them pristine, whole, and untouched, and then were washed and air-dried. The arils were sown in peat moss pads to grow young seedlings.

### RNA extraction

Total RNA from 10-day-old seedlings was extracted as described [38] by using TRIzol reagent (Invitrogen, Carlsbad, CA) and the RNA MiniPrep kit (Zymo Research, Irvine, CA). Total RNA from leaves, flowers and fruits of different developmental stages was extracted using a modified CTAB-LiCl method [39]. For fruit samples, we used only separated arils for all developmental stages. About 200 mg of finely ground sample in liquid nitrogen for each tissue was used for extraction. Extraction buffer I, II and other solutions were prepared as suggested [39]. The chloroform: isoamyl alcohol (24:1) and LiCl steps were repeated three times. Finally, the RNA pellet was dissolved in 40 μL RNase-free water. All RNA samples were purified with use of the RNA Clean & Concentrator kit with on-column digestion of genomic DNA by using DNase I (Zymo Research, Irvine, CA). RNA integrity number > 8.0 was confirmed by use of the 2100 Bioanalyzer (Agilent Technologies, Santa Clara, CA). For global miRNA transcriptome profiling, an equimolar concentration of total RNA extracted from three biological replications of all samples was pooled and sent for RNA sequencing. Total RNA from all three biological replications was independently used in stem-loop RT-qPCR.

### Small RNA sequencing

sRNA samples were sequenced by the Beijing Genomics Institute (BGI, Hong Kong) with the Illumina HiSeq 2000 platform. The construction of the sRNA library and sequencing consisted of the following steps [40]. After extracting the total RNA from the samples, sRNAs of 18 ~ 30 nt were gel-purified, 5’ RNA adapter-ligated and gel-purified, 3’ RNA adapter-ligated and gel-purified, then underwent RT-PCR and gel purification. Finally, the library products were ready for sequencing by using Illumina HiSeq 2000.

sRNAs from deep sequencing covered almost every kind of RNA, including miRNAs, siRNAs, piwi-interacting RNAs (piRNAs), ribosomal RNAs (rRNAs), transfer RNAs (tRNAs), small nuclear RNAs (snRNAs), small nucleolar RNAs (snoRNAs), repeat-associated sRNAs and degraded tags of exons or introns. The sRNA digitization analysis based on high-throughput sequencing involved use of sequencing by synthesis (SBS), which can decrease the loss of nucleotides caused by the secondary structure. This HiSeq method is robust and also strong because of its requirement for small sample quantity, high throughput, and high accuracy with a simply operated automatic platform. Such analysis resulted in millions of sRNA sequence tags from the pomegranate RNA sample.

### RNA-seq bioinformatics analysis and miRNA prediction

After sequencing, raw sequence reads (FASTQ files) were processed into clean reads, then filtered to discard low-quality adapter contaminative tags, and the remaining reads with lengths < 18 nt were discarded. Usually, the sRNA is 18 to 30 nt (miRNA, 21 or 22 nt; siRNA, 24 nt; and piRNA, 30 nt). All unique clean reads, specifically non-redundant ones, were considered for further analysis, including non-coding RNA identification and proper annotation. First, clean reads of sRNAs such as rRNAs, small cytoplasmic RNAs (scRNAs), snoRNAs, snRNAs, and tRNAs were identified by a BLASTall search against the Rfam (v10.1) and GenBank databases. miRNAs were identified by mapping sRNA reads against poplar genome sequences by using SOAP2 [41]. The SOAP2 output was filtered with use of in-house filter tool to identify the candidate sequences as miRNA precursors by analyzing a mapping pattern of one or more blocks of aligned small RNAs with perfect matches [42]. The secondary structures of candidate sequences were checked by applying stringent criteria as suggested [43]. To determine conserved miRNAs, clean reads were compared with known plant miRNAs deposited at miRBase [44]. Those with non-perfect matches were considered variants of known miRNAs. Other sequences that did not map to known miRNAs and other kinds of sRNAs were considered un-annotated sequences for novel miRNA prediction. To obtain the miRNA predicted precursor structure, the sequences were analyzed by using TurboFold [45] http://rna.urmc.rochester.edu/RNAstructure.html) and guide and star sequences were obtained.

### Target prediction, functional annotation and pathway analysis

The target prediction method involved loading miRNA reads in a FASTA format file containing sRNA sequences to search for targets from a known poplar (*Populus trichocarpa*) transcript database by using the suggested rules [46, 47]. Specifically, criteria for choosing miRNA/target duplexes were 1) less than four mismatches between sRNA and the target, 2) less than two adjacent mismatches in the miRNA/target duplex, 3) no adjacent mismatches in positions 2–12 of the miRNA/target duplex (5’ of miRNA), 4) no mismatches in positions 10–11 of the miRNA/target duplex, 5) less than 2.5 mismatches in positions 1–12 of the miRNA/target duplex (5’ of miRNA), and 6) minimum free energy (MFE) of the miRNA/target duplex ≥74 % of the MFE of the miRNA bound to its perfect complement. To investigate the putative functions of potential target genes, the target sequences from poplar were annotated by using the databases Gene Ontology (GO) and Kyoto Encyclopedia of Genes and Genomes (KEGG) Orthology (KO) [48, 49]. The GO results were classified into three independent groups: cellular component, molecular function, and biological process. KO pathways were grouped into different metabolism functions and signal transduction.

### Validation of miRNA variants and novel miRNAs by stem-loop RT-qPCR

Stem-loop RT-qPCR was used to confirm the differential expression of miRNAs and their variants across leaves, flowers, and fruit developmental stages. About 1 μg DNA-free total RNA was hybridized with miRNA-specific stem-loop RT primers for six miRNA families and six novel miRNAs, and the hybridized molecules were reverse-transcribed into cDNAs with use of the Superscript III kit (Thermo Fisher Scientific, Waltham, MA USA). The forward miRNA-specific primer for the mature miRNA sequences and the universal reverse primer for the stem-loop sequences were designed (Additional file 1: Table S8). For each reaction, 1 μL cDNA, 10 μL 2X FastStart SYBR Green (Roche), and primers were mixed. PCR runs were 95 °C for 10s, 60 °C for 30s with the StepOnePlus Real-Time PCR System (Applied Biosystems, Foster City, CA, USA). The expression of miRNAs was normalized to that in leaves in all three biological replications. 5.8S ribosomal RNA was used as reference to calculate relative gene expression by the 2^-ΔΔCt^ method [50].

## Results and discussion

Pomegranate fruit contains a variety of natural compounds such as phenolics, alkaloids, terpenoids, and fatty acids and has a role in numerous health-promoting activities [51]. Both fruit peels and arils are used to extract natural compounds such as punicalagin (derivative of gallic acid and glucose) and anthocyanins (class of water-soluble phenolic compounds responsible for the pink to red fruit) [52]. Many reports describe the benefits of pomegranate natural products for humans, but lack of genomic information is a major bottleneck in genomic research of pomegranate. In this study, we profiled the conserved and novel miRNAs in pomegranate and discuss their different biochemical pathways.

### Fruit development and collection of tissues

Pomegranate fruit development is divided into different stages. The fruit growth pattern depends on the cultivar as well as location and season [53, 54]. We divided the developmental stages of Al-sirin-nar as follows (Fig. 1): stage 1, approximately 8–10 days from initial flowering (petal drop stage); stage 2, approximately 10 days from stage 1 (fruit has begun to expand, but no color change); stage 3, approximately 12–15 days later (fruit has swelled more and is just starting to change from red to green); stage 4, approximately 15–18 days later (fruit has expanded from pear shape to more rounded shape, more green from red); stage 5, approximately 15 days later (continued expansion of fruit, color continues to change from red to green); and stage 6, approximately 15 days later (continued expansion of fruit, color continues to change from red to green), the calyx remains red, referred to as the “lipstick” stage. The process takes 75 to 85 days from initial flowering to stage 6. After stage 6, the fruit becomes glossy red and contains rosy-pink arils with a sweet tart taste. To profile the overall miRNA expression, we collected leaves, male and female flowers and fruit tissues from different stages. Throughout the fruit developmental stages, the color development of the peel (fading of dark red) and arils inside the fruit (accumulation of dark red) is the reverse. So, the anthocyanin is increasingly accumulating in arils during the later stages of fruit development.

During fruit development, pomegranate accumulates a variety of phytochemical compounds [55] that function as a defense mechanism. The edible part is 50 % of the fruit: 40 % and 10 % are arils and seeds, respectively. Arils contain mostly water (85 %), 10 % sugar (glucose and fructose), organic acids (citric acid, ascorbic acid, malic acid), and the bioactive compounds anthocyanins (phenolics and flavonoids) [56]. In addition, the seed cover contains six types of glucosides, with delphinidin-3,5-diglucoside the main anthocyanin in juice [57]. Pigmentation of fruit peel and arils is an important quality indicator of fruit. Al-sirin-nar fruit peel is rosy-red as compared with dark red for ‘Wonderful’, and the color of peel and arils is not related [2].

### High-throughput sequencing and annotation of small RNAs

Total RNA was extracted from young seedlings and other tissues and pooled for building a small RNA library for further sequencing. About 30 million reads were generated by using Illumina HiSeq 2000 (Table 1). From 29.95 million high-quality reads after removing 5’- and 3’ adapters, insert nulls, sRNAs < 18 nt, and poly A reads, 99 % clean reads was obtained. A total of 8,603,217 (28.97 %) reads in all categories were unique to pomegranate. Because the genome sequence of pomegranate is not available and poplar is a deciduous flowering tree with full genome information, we used the poplar genome as a reference for mapping the clean reads with use of SOAP2 [41].

**Table 1:** Overview of miRNA sequencing reads

Read type	Count	Percent
Total reads	30000000	-
High-quality reads	29948480	100.00 %
3’adapter-null reads	43776	0.15 %
Insert-null reads	5228	0.02 %
5’adapter contaminants	92262	0.31 %
<18-nt reads	115751	0.39 %
PolyA reads	1429	0.00 %
Clean reads	29690034	99.14 %

**Fig. 2:**
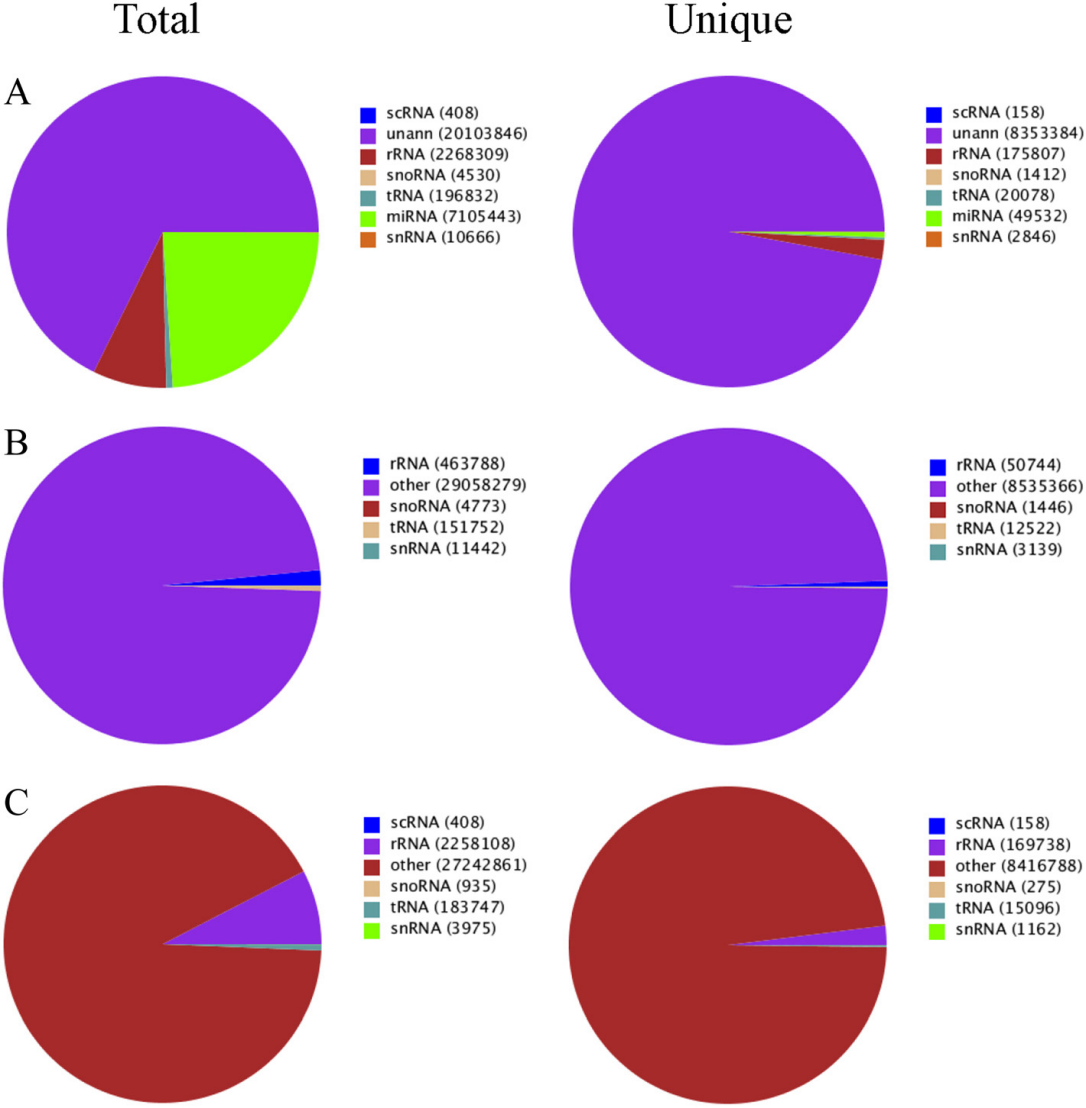
Distribution of small RNAs by annotation categories. Pie chart shows pomegranate small RNAs matching data in the non-coding RNA database. **a** Cumulative distribution of different non-coding small RNA categories pooled from Rfam and NCBI. **b** Small RNAs matching Rfam non-coding RNAs. **c** Small RNAs matching GenBank non-coding RNAs. Each small RNA database shows differences in subcategories depending on the availability of existing data

**Fig. 3:**
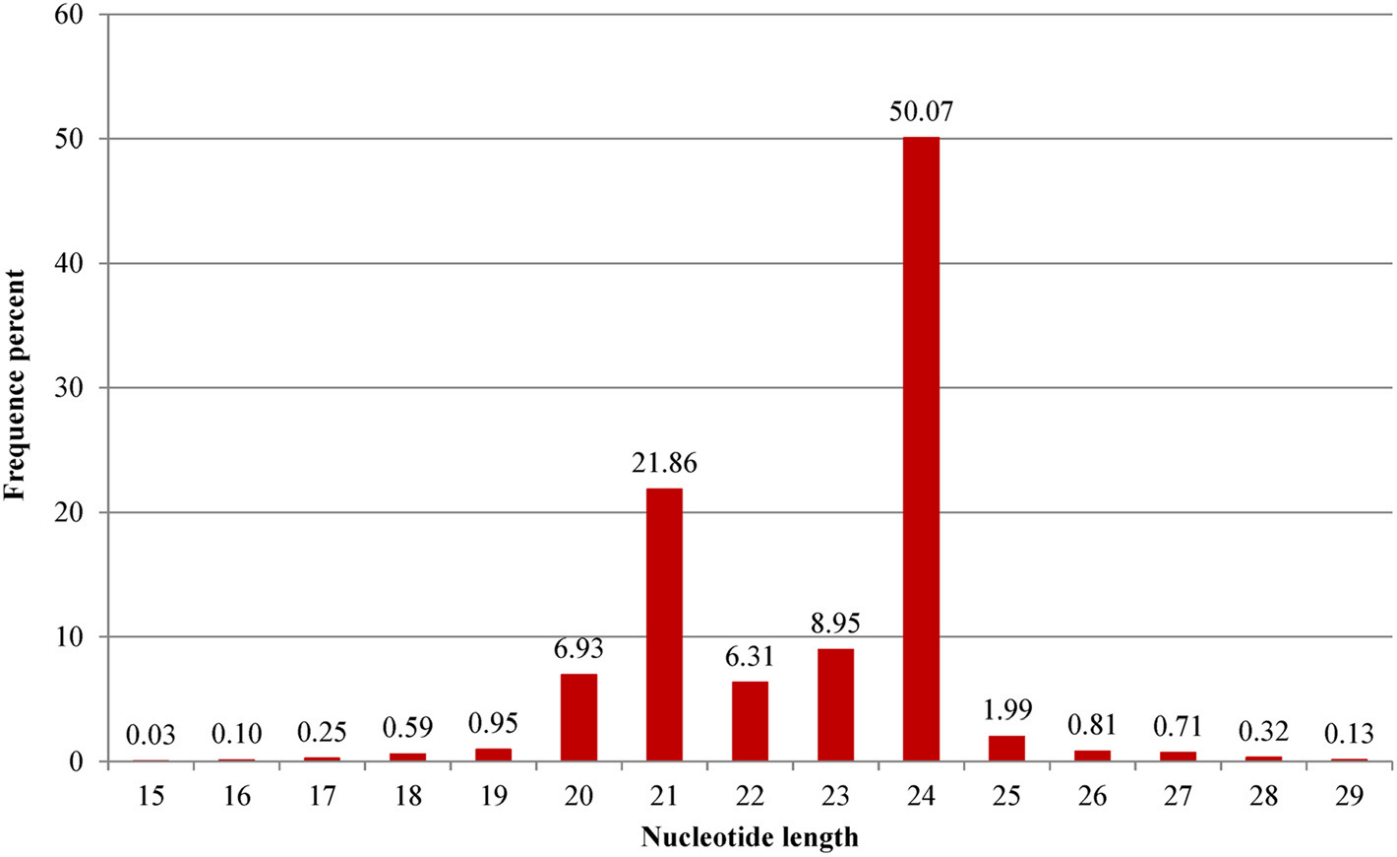
Length distribution of all small RNA tags

Approximately 8.3 % (2,480,745) of the reads were mapped to the known non-coding RNAs, including scRNAs, tRNAs, snoRNAs, and snRNAs (Fig. 2a). Among all sRNAs, 23.9 % belonged to miRNAs, 0.7 % unique to pomegranate. However, the number of reads in each category differed when matched to the Rfam and GenBank databases. Particularly, the number of rRNA specific reads was high (2,258,108) in GenBank but low (463,788) in Rfam. The number of other sRNAs, including miRNAs, was more or less similar in both Rfam and Genbank databases (Fig. 2b, c). Most of the known and novel small RNA reads identified in pomegranate were 24 nt (~50 %), followed by 21 nt (21.8 %), 23 nt (8.95 %), 20 nt (6.93 %), and 22 nt (6.31 %). Other sRNAs between 15 and 29 nt were not significantly abundant (Fig. 3; Additional file 2: Table S1). The 24-nt small RNAs also exist in many plant species such as maize, Arabidopsis, tomato, barrel clover, and trifoliate oranges [40, 58–61]. Thus, the 24-nt small RNAs may also be involved in critical functions in pomegranate as in other plants.

### Identification of conserved miRNAs in pomegranate

miRNAs in plant systems can be identified by examining the potential fold-back precursor structure containing a ~21-nt sequence within one arm of the hairpin structure. To identify the known miRNAs and obtain miRNA counts, we used the base bias on the first position of identified miRNAs and on each position separately in the pomegranate library; clean reads of sRNA tags were aligned to the miRNA precursor/mature miRNA of plant and animals deposited in miRBase 20.0 (http://www.mirbase.org) [62]. The results gave information on alignment, including the structure of known miRNA precursors, lengths and counts. A total of 30 known miRNA families from our library matched miRBase, containing 28,645 entries (Table 2). Analysis of read counts for known miRNA families indicated that the expression frequency varied significantly from 4,015,427 to 511 among different miRNA families. Known miRNA families with less than 500 reads were ignored. Each miRNA family featured various counts with its own variants. MiR157 was the most abundant family (4,015,427) followed by miR156 (1,632,172), miR166, miR168, miR167, miR535, miR169, and miR390. The number of variants of well-known miRNAs in pomegranate was high for miR156 followed by miR157, miR159 and miR160. These miRNAs showed variation for a few families in pomegranate despite high counts (Additional file 3: Table S2).

**Table 2:** Details of conserved miRNA families in pomegranate

miRNA family	Counts	Sequence	miRBase database
miR157	4015427	TTGACAGAAGATAGAGAGCAC	ath-miR157a
miR156	1632172	TGACAGAAGAGAGTGAGCAC	csi-miR156
miR166	454425	TCGGACCAGGCTTCATTCCCC	pvu-miR166a
miR168	299953	TCGCTTGGTGCAGGTCGGGAA	ath-miR168a-5p
miR167	159932	TGAAGCTGCCAGCATGATCTGA	ccl-miR167a
miR535	104111	TGACAACGAGAGAGAGCACGC	ppt-miR535a
miR169	84700	TGAGCCAAGAATGACTTGCCGG	cme-miR169t
miR390	33324	AAGCTCAGGAGGGATAGCGCC	ath-miR390a-5p
miR479	19760	CGTGATGTTGGTTCGGCTCATC	ghr-miR479
miR171	10381	CGAGCCGAATCAATATCACTC	csi-miR171b
miR2916	8924	GGGGCTCGAAGACGATCAGATA	peu-miR2916
miR482	4825	TTCCCAAGGCCGCCCATTCCGA	mdm-miR482a-3p
miR160	4710	GCGTATGAGGAGCCATGCATA	ptc-miR160b-3p
miR4414	4472	TGTGAATGATGCGGGAGATAC	mtr-miR4414b
miR159	4088	TTTGGATTGAAGGGAGCTCTA	ptc-miR159a
miR164	2755	TGGAGAAGCAGGGCACGTGCA	ptc-miR164a
miR6300	2507	GTCGTTGTAGTATAGTGGT	gma-miR6300
miR319	2113	TAGCTGCCGACTCATTCATCCA	ppe-miR319b
miR894	1830	GTTCGTTTCACGTCGGGTTCACCA	ppt-miR894
miR408	1620	CTGGGAACAGGCAGGGCATGG	ptc-miR408-5p
miR172	1608	AGAATCTTGATGATGCTGCAT	ptc-miR172a
miR162	1581	TCGATAAACCTCTGCATCCAG	ptc-miR162a
miR396	1566	GCTCAAGAAAGCTGTGGGAAA	ath-miR396b-3p
miR3639	1389	AAATGACTTCTGAACGGCAAAAC	vvi-miR3639-5p
miR6248	792	TAATTGTGGATGGAGGTAT	osa-miR6248
miR1171	721	TGGGAATGGAGTGGAGTGGAGTAG	cre-miR1171
miR858	658	TTCGTTGTCTGTTCGACCTTG	ath-miR858b
miR5653	533	TGAGAGTTGAGTTGAGTTGAGTTT	ath-miR5653
miR530	513	TGCATTTGCACCTGCACCTTA	ptc-miR530a
miR4415	511	AAGGTTGTGATTGGAATTAATGGC	gma-miR4415b-5p

Because of their high sequence similarity and conserved targets, miR156 and miR157 were grouped into a single family. Cleavage of the Squamosa promoter binding protein-like (SPL) by miR156/157 has been confirmed in different crops including Arabidopsis [63] and rice [64, 65]. In our studies, miR157 was the largest miRNA family among all families. This finding contrasts with recent reports of pear fruit development [66] and peanut [67], showing miR156 as the most abundant. MiR157 may have unique targets and common targets between miR156/miR157. In addition to families, variants of each family showed differential expression. The number of miRNAs was counted and normalized to total reads of sRNAs. The total counts for each family variant varied greatly. The expression of miRNA families of miR157a, miR156, miR157b, miR156a, miR156g, miR159a and miR160b was high in our pooled pomegranate sample. In contrast, a few other families and variants showed less expression (Additional file 3: Table S2). The abundance of each family also varied. When the miRNAs were predicted from miRBase, different family members exactly matched known miRNAs from different plants such as Arabidopsis, rice, grapes, poplar tree, maize, and soybean.

### Novel miRNAs and their identification in pomegranate

**Fig. 4:**
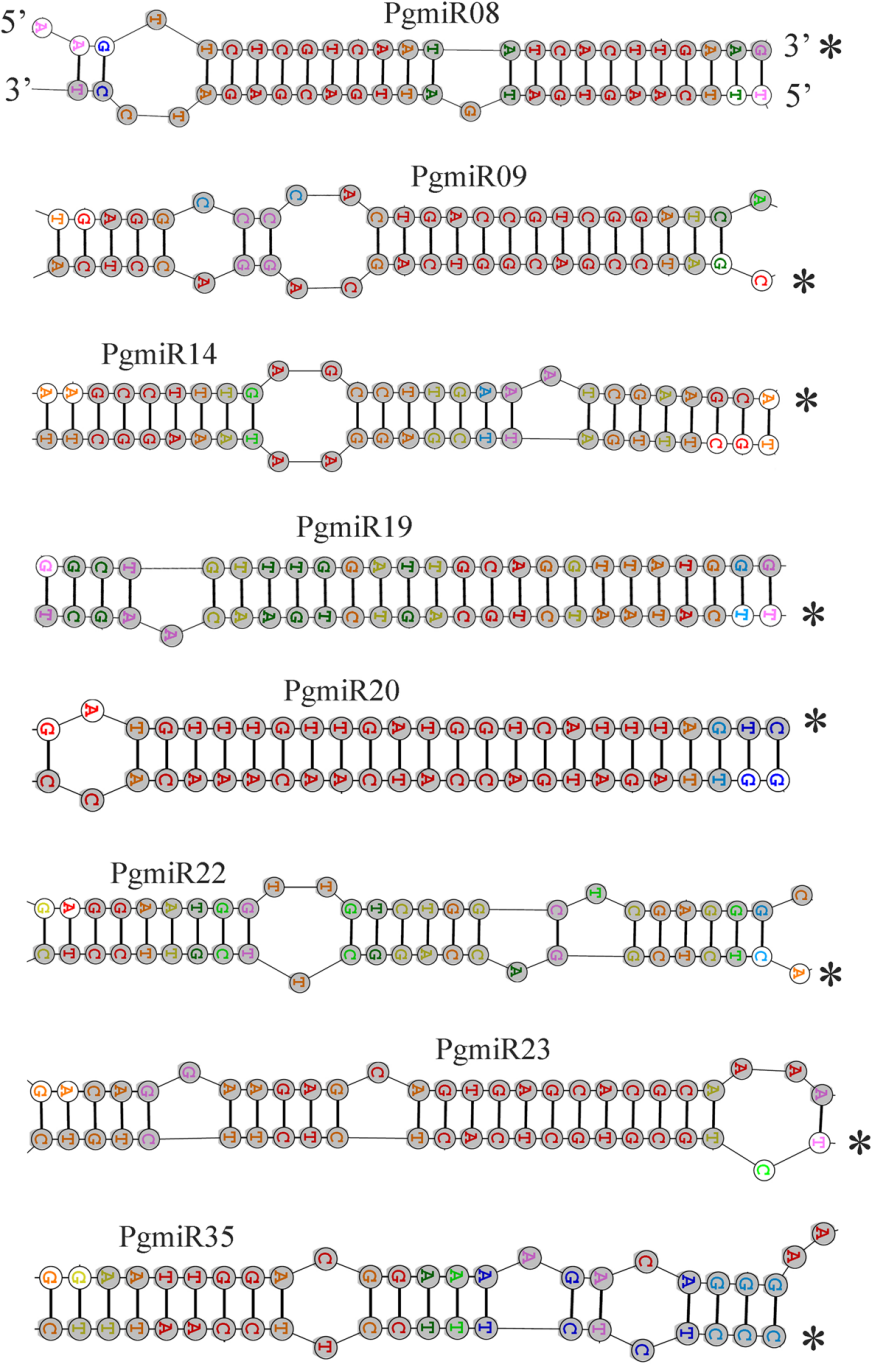
Predicted precursor structures of novel miRNAs found in pomegranate. The predicted fold-back structures of few selected miRNAs precursors from novel miRNA pool based on minimum folding free energy. The regions of miRNA are shaded with grey color. The miRNA guide strand is marked with asterisk

To reveal the novel miRNA candidates from the pomegranate small RNA library, we used MIREAP and explored the characteristic hairpin structure of miRNA precursors. Only secondary structures with the lowest free energy and a high degree of pairing were included as miRNA precursors. Precursors forming hairpin structures for the 10 novel miRNAs (Table 3) were predicted with an average minimum folding free energy of − 55.82 kcal/mol, from − 73.1 to − 31.94 kcal/mol (Additional file 4: Table S3). The counts of novel miRNAs ranged from 115 (PgmiR25) to 4807 (PgmiR08). The length of precursors of the novel miRNAs ranged from 74 nt (PgmiR35) to 336 nt (PgmiR20). This length range is almost similar to novel miRNA precursors of Japanese apricot [68]. Among 10 miRNAs, 6 had a 5’ arm and 4 had a 3’ arm. The stem loop structures of predicted novel miRNA candidates were drawn from the precursor sequences by using TurboFold (Fig. 4) [45].

**Table 3:** Predicted novel miRNAs in pomegranate

miRNA ID	Count	Seq (5p)	Seq (3p)
PgmiR08	4807	-	TCAAGTGATGATTGACGAGATC
PgmiR09	852	AGGCCCCACTGACCGTCGGAT	-
PgmiR14	358	-	TTTGATTCGAGGAATAAAGGC
PgmiR19	245	CTGTTTGGATTGCAGGTTATG	-
PgmiR20	102	-	TTAGATGACCATCAACAAACA
PgmiR22	1615	GGAATGGTTGTCTGGCTCGAGG	-
PgmiR23	323	CAGGAAGAGCAGTGAGCACGCAA	-
PgmiR25	115	GAAGCTGACGAGGGAGAGTGG	-
PgmiR31	255	-	TACTAGCTGTAGGGATATTGC
PgmiR35	1350	AATTGGACGGAAAAGACAGGG	-

**Fig. 5:**
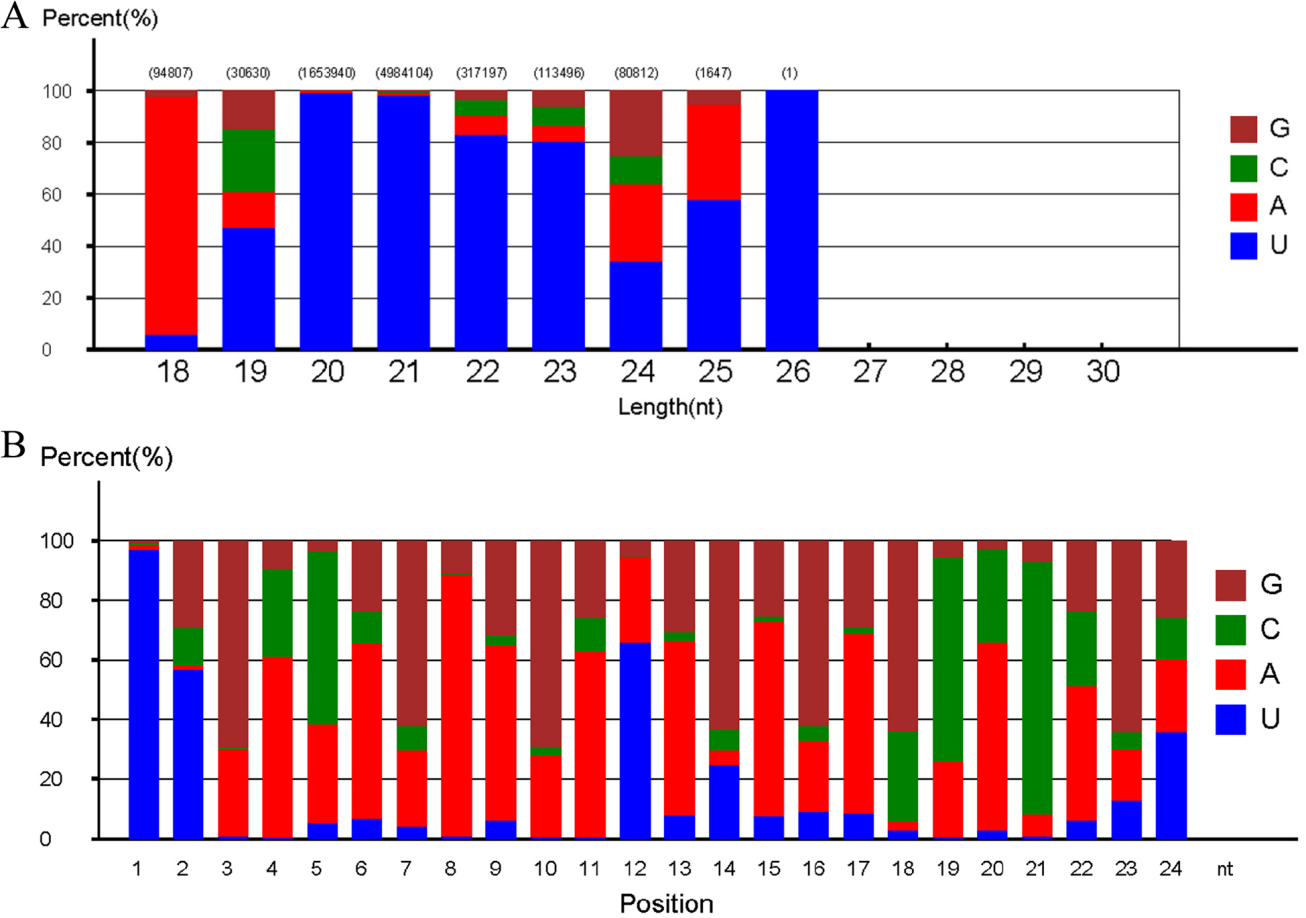
miRNA variants and their nucleotide bias position. **a** First nucleotide bias for the first position of 18- to 26-nt miRNAs. Nucleotide U predominates. **b** MiRNA nucleotide bias for each position of 24-nt miRNAs

Novel miRNA prediction was summarized as the base bias on the first position from the 5’ end and base bias on each position (Fig. 5). With the exception of 18 nt for 18- to 26-nt small RNAs, the base bias at the first position from the 5’ end had a strong preference for U but not G. Nucleotides A and U predominately occupied the first position base bias for the 18- and 20-nt small RNAs, respectively, which agreed with the base bias results for *Acipenser schrenckii* [69].None of the miRNAs in this range showed a G or C preference (Fig. 5a; Additional file 5 Table S4). Even though nucleotide U was preferred more than 80 % of the time as a first base for 20- to 23-nt mRNAs, base biases for 21- to 23-nt novel miRNAs showed a pattern of U followed by A, C and G in the pomegranate library. For nucleotide bias at each position of 24-nt mRNAs, overall, nucleotide A was the most prevalent (37.7 %), followed by G (30.3 %), C (17.0 %), and U (15.0 %). The proportion with U at the first and second positions was 96.9 % and 56.9 %, respectively, which was similar to golden-thread orchid [70]. The predominant positions of bases in 24-nt sRNA tags from position 1 to 24 were UUGACAGAAGAUAGAGAGCACAGU (Fig. 5b; Additional file 6: Table S5).

### Validation of high-throughput RNA-sequencing in different tissues

**Fig. 6:**
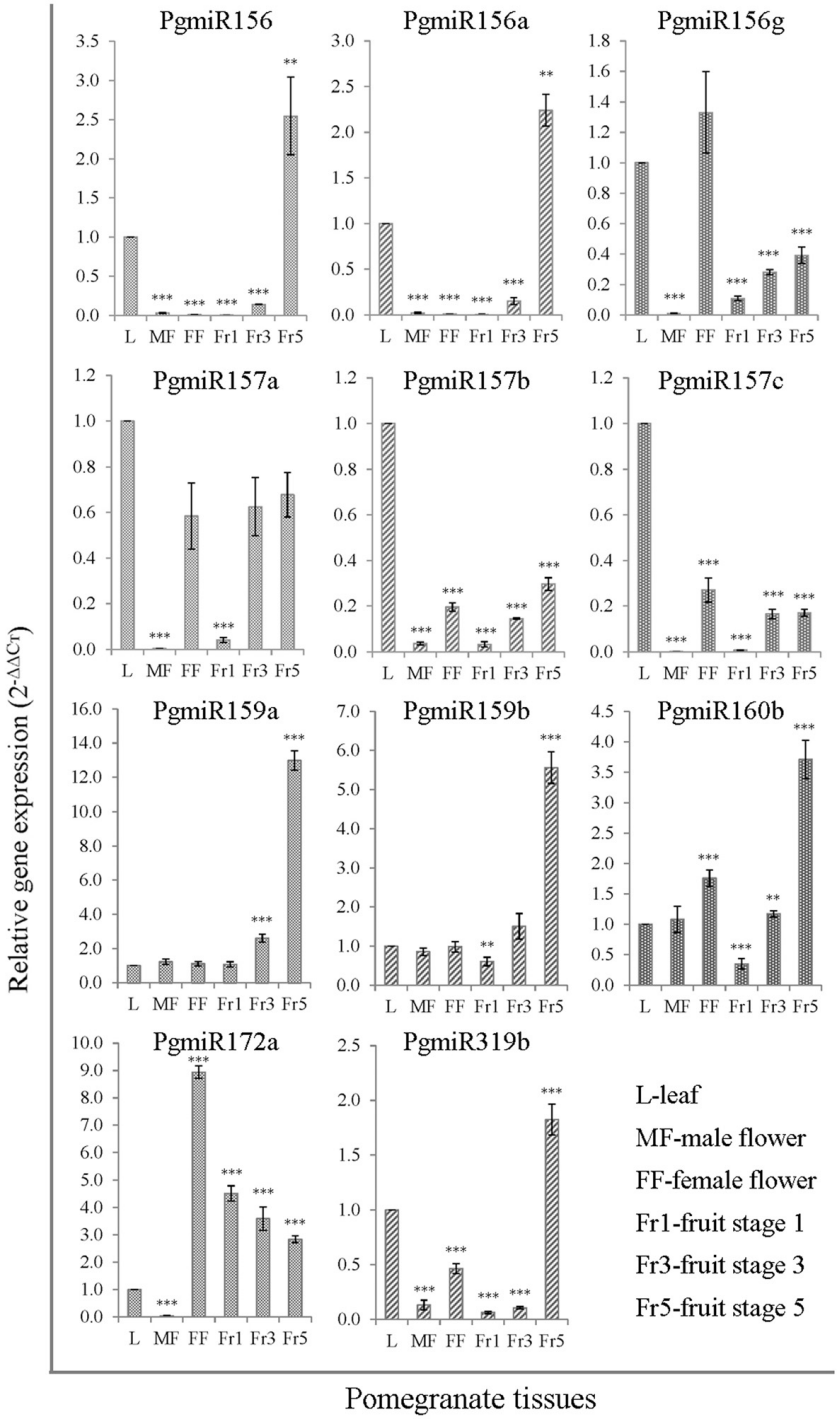
Stem-loop RT-qPCR validation of highly expressed known miRNAs and their variants in different tissues. Relative quantity is based on the expression of the reference gene 5.8 s ribosomal RNA. X-axis indicates different tissues and Y-axis the expression of miRNA relative to that in leaf tissue. Data are mean ± SD from three biological replicates. **, *P* < 0.01; ***, *P* < 0.001 by Student *t* test. Bar values higher or lower compared to leaf tissue indicates upregulation or downregulation, respectively

**Fig. 7:**
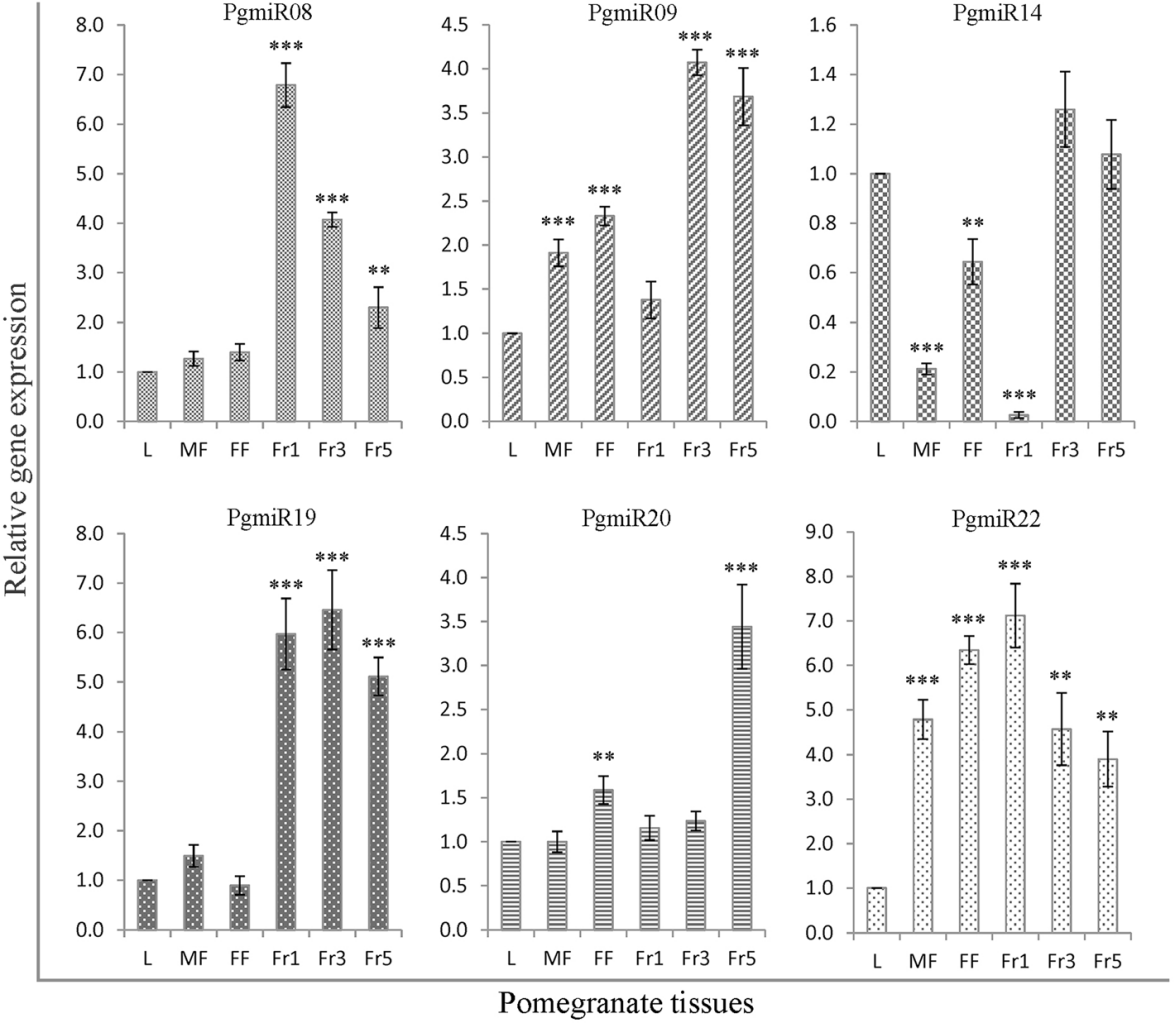
Stem-loop RT-qPCR validation of novel miRNAs in various pomegranate tissues. Relative quantity is based on expression of the reference gene 5.8 s ribosomal RNA. X-axis indicates different tissues (L, leaf; MF, male flower; FF, female flower; Fr1, fruit stage 1; Fr3, fruit stage 3; Fr5, fruit stage 5) and Y-axis indicates the expression of miRNA relative to that in leaf tissue. Data are mean ± SD from three biological replicates. **, *P* < 0.01; ***, *P* < 0.001 by Student *t* test. Bar values higher or lower compared to leaf tissue indicates upregulation or downregulation, respectively

To elucidate the potential roles of miRNAs in pomegranate fruit development, we profiled the expression levels of known and novel miRNAs. miRNAs have wide expression in plant tissues and play multiple key regulatory roles in physiological and developmental processes [71]. Most miRNAs in plants regulate developmental processes by destroying their target mRNAs because the target gene has complete complementarity with miRNA [72]. We used stem-loop RT-qPCR with unique primer sets to validate the expression pattern of highly expressed miRNA families (PgmiR156, PgmiR157, PgmiR159, PgmiR160, PgmiR172, and PgmiR319) and their variants (PgmiR156a, PgmiR156g, PgmiR157b, PgmiR157c, and PgmiR159b) in leaves, male and female flowers, and different fruit developmental stages of pomegranate (Fig. 6). This method could confirm the existence of pomegranate miRNAs and also detect the expression of miRNAs in various tissues. We found a differential expression pattern across tissues. The miRNAs PgmiR156, PgmiR156a, PgmiR159a, PgmiR159b, PgmiR160b, and mPgiR319b were highly upregulated during later stages of fruit development; that of PgmiR172 was high in female flowers, then gradually decreased to a lower level with fruit maturity. Other family members were ubiquitously expressed in leaves and other tissues including fruits. Additionally, we validated a few novel miRNAs with high count reads from sequencing (Fig. 7). Their expression pattern differed among tissues. Novel miRNAs PgmiR08 and PgmiR22 showed high expression during the early developmental stage of fruit that receded toward the final maturity stage. Interestingly, PgmiR19 appeared to express only during fruit developmental stage and not in leaf and flower. These differentially expressed miRNAs may regulate different targets during fruit development and ripening.

### Prediction of miRNA target genes, gene ontology (GO) and KEGG pathway analysis

Most of the targets of miRNA are conserved across several plants including Arabidopsis, rice, poplar, and wheat [73–76]. Majority of them are various transcription factors including SQUAMOSA promoter binding protein-like (SPB/SPL) (miR156), NAM (miR164), MYB (miR159, miR172, miR319) that regulate plant development and phytohormone signaling [77]. SPL is one of the miR156 targets in Arabidopsis [78], with expression inversely related to that of miR156. SPL, which shows abundant expression in the absence of miR156 expression in early stages of fruit development, may destabilize the MYB-bHLH-WD40 complex to repress the anthocyanin biosynthetic pathway and further accumulation [64]. Keeping this hypothesis in mind, with increased SPL expression being a negative regulator of anthocyanin accumulation, the anthocyanin content in pomegranate might be still under the detectable level with increased flavonol quantity in early aril developmental stages. However, SPL expression may be decreased during later stages of maturity to accumulate anthocyanin with increased PgmiR156 expression. Although this conclusion is premature without quantifying SPL accumulation in different fruit stages, the increased expression of PgmiR156/PgmiR157 we observed might have a positive effect on increasing anthocyanin and proanthocynidin or tannin levels in mature pomegranate.

To better understand the functions of identified novel miRNAs in pomegranate, we predicted putative candidate genes by using bioinformatic analyses [79, 80]. A total of 288 target genes were identified for ten novel miRNAs and gene ontology with annotation details have been found (Additional file 7: Table S6). Consistent with previous reports, most of the novel miRNA targets belong to plant-specific transcription factors, (AP2, MYB, ARF, GRAS, PHD, and bZIP), followed by regulators of metabolic processes (protein kinases, LRR kinase, RLKs, etc.) and hormone signaling. In addition, there are several other targets whose functions are largely unknown. The targets of PgmiR08 ARFs, bHLH, SecY protein, TIR1 F-box, and auxin signaling F-box2 (AFB2) are shown to be involved in root and fruit development, anthocyanin accumulation as well as in abiotic stress. In contrast to climacteric fruits (apple, banana, tomato), notably little is known about the hormonal control of ripening in non-climacteric fruits such as pomegranate, grape, and strawberry [81] and it has been proven that even ethylene levels or respiration was considerably low during ripening of non-climacteric fruit [82]. That could be one possibility that we did not find any major ethylene pathway candidates in our target identification. Anthocyanin biosynthesis is a branch of the flavonoid pathway and genes involved in anthocyanin biosynthesis and regulation have been discovered and studied in several fruits, such as bHLH in apple [83], and MYB and bHLH in peach [84]. To support this notion, ARF10 plays key role in anthocyanin biosynthesis of pomegrante. The GO (Additional file 7: Table S6) shows that MYB transcription factor, the target of PgmiR14, PgmiR22 and PgmiR31 is involved in multiple hormone signaling including gibberellic acid, ethylene and salicylic acid during fruit development and ripening [85].

In addition, GRAS transcription factor (PgmiR25), and nuclear transcription factor Y (PgmiR22), copper transporter (PgmiR09), disease resistance protein TIR-NBS-LRR, and LRR protein kinase (PgmiR31) are the targets of few novel miRNAs. Recently, genes coding for GRAS transcription factors were identified as targets of miRNAs during fruit development and ripening of tomato [86] and grapevine [87]. Moreover, F-box family proteins play vital roles in the signal transduction pathways of different hormones [88] and 166 F-box genes were identified during maturation and fruit ripening in apple [89]. Group of F-box genes targeted by PgmiR08 and PgmiR20 might participate mostly in auxin signaling pathway towards fruit ripening. During fruit development, synthesized sucrose in the leaf is transported to sink tissues such as fruits where it is directly used for metabolism or translocated into storage tissues for the synthesis of major storage products through carbohydrate metabolism [90]. Mutants of sucrose transporters (SUT) exclusively affected tomato fruit and seed development [91]. SUT2, the target of PgmiR31 and the key player in sucrose:hydrogen symporter activity, might be a key player in normal fruit development. The seed development is part of fruit maturity and ripening, and the development of both occurs simultaneously. In pomegranate, seeds which are inside the arils are surrounded by juice. A nuclear transcription factor Y subunit A-1 (NF-YA1) targeted by novel miRNAs PgmiR22 and PgmiR23, and a bZIP transcription factor targeted by PgmiR31 seem to involve in seed maturation and dormancy in the arils of pomegranate fruits.

Above all, tissue integrity and cation binding to the cell wall during fruit senescence is very important, and pectin methylesterase (PME) activity modifies tissue integrity in ripening tomato [92]. As an ubiquitous plant enzyme, PME catalyzes the deesterification of galactosyluronate methyl esters of pectin to their free carboxylic groups, and has been suggested to cause transesterification to uronoyl-sugar crosslinks [93]. PME has been implicated in various processes in ripening fruits including textural changes, formation of abscission zones and cell wall growth, maturation, and extensibility. Alongside, invertases may involve in the long-distance transport of sucrose and take part in phloem loading and unloading [94]. From our transcriptome and GO analysis, we believe that plant invertase/pectin methylesterase inhibitor could be targeted by PgmiR31 to aid in fruit ripening process. Overall, the known and unknown targets of novel microRNAs participate in pomegranate fruit development and further ripening process.

**Fig. 8:**
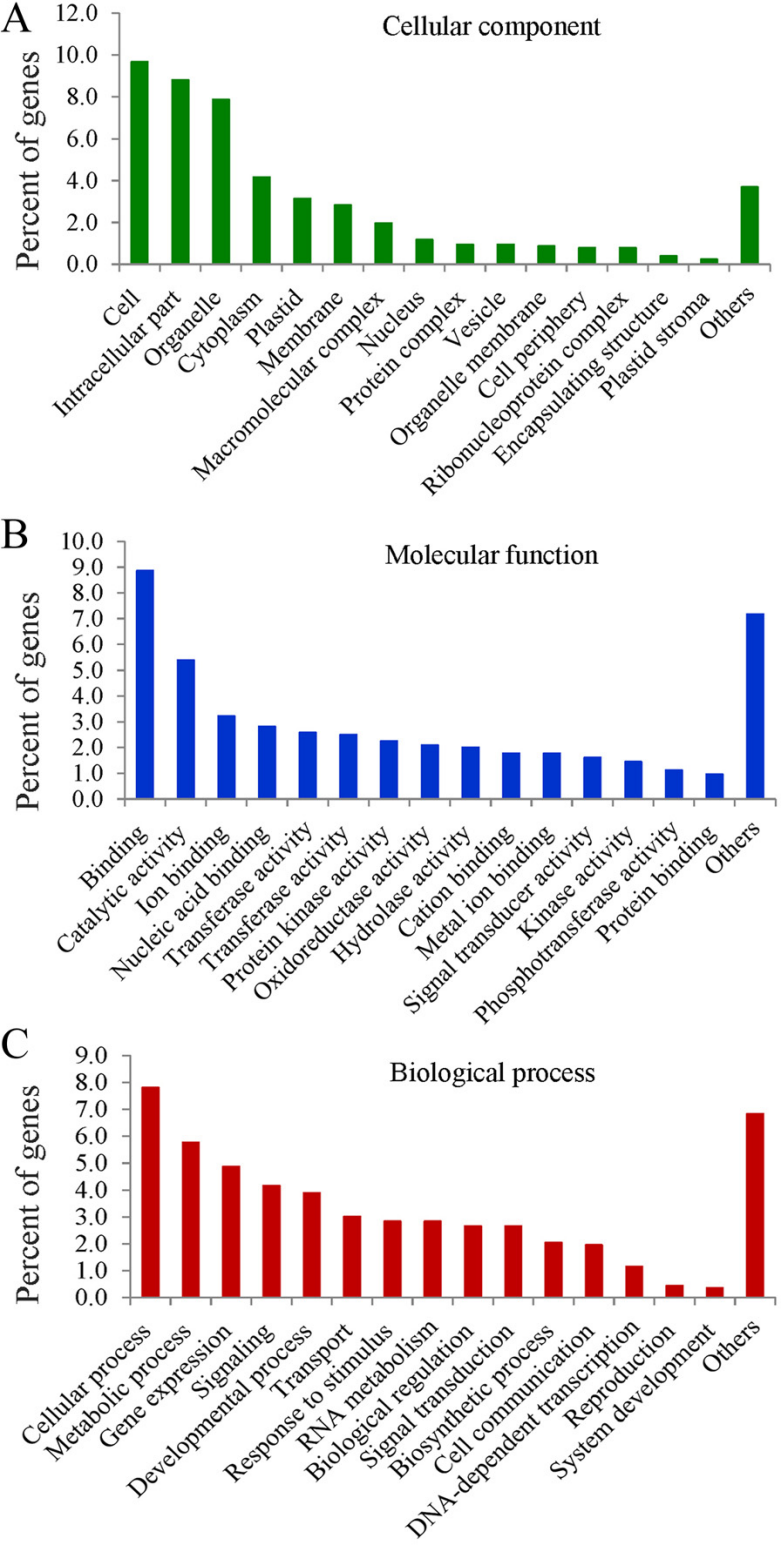
Gene ontology categories for miRNA targets in pomegranate. Target genes were classified into the categories cellular component (**a**), molecular function (**b**), and biological processes (**c**). Values in the Y axis are the percentage of target genes in different functional categories

To evaluate the potential functions of the miRNA target genes, GO categories were assigned to all of the predicted genes, which resulted in three unique categories: cellular component, molecular function, and biological processes (Fig. 8). In the cellular component, the major categories were “cell,” followed by “intracellular part” and “organelle”. In the molecular function category, the major categories were “binding” and “catalytic activity.” For biological process, the “cellular” and “metabolic processes” were the most abundant categories. Metabolic processes are the key active process in fruit development [66], and cellular processes and metabolic processes were the top two GO categories within biological processes.

**Fig. 9:**
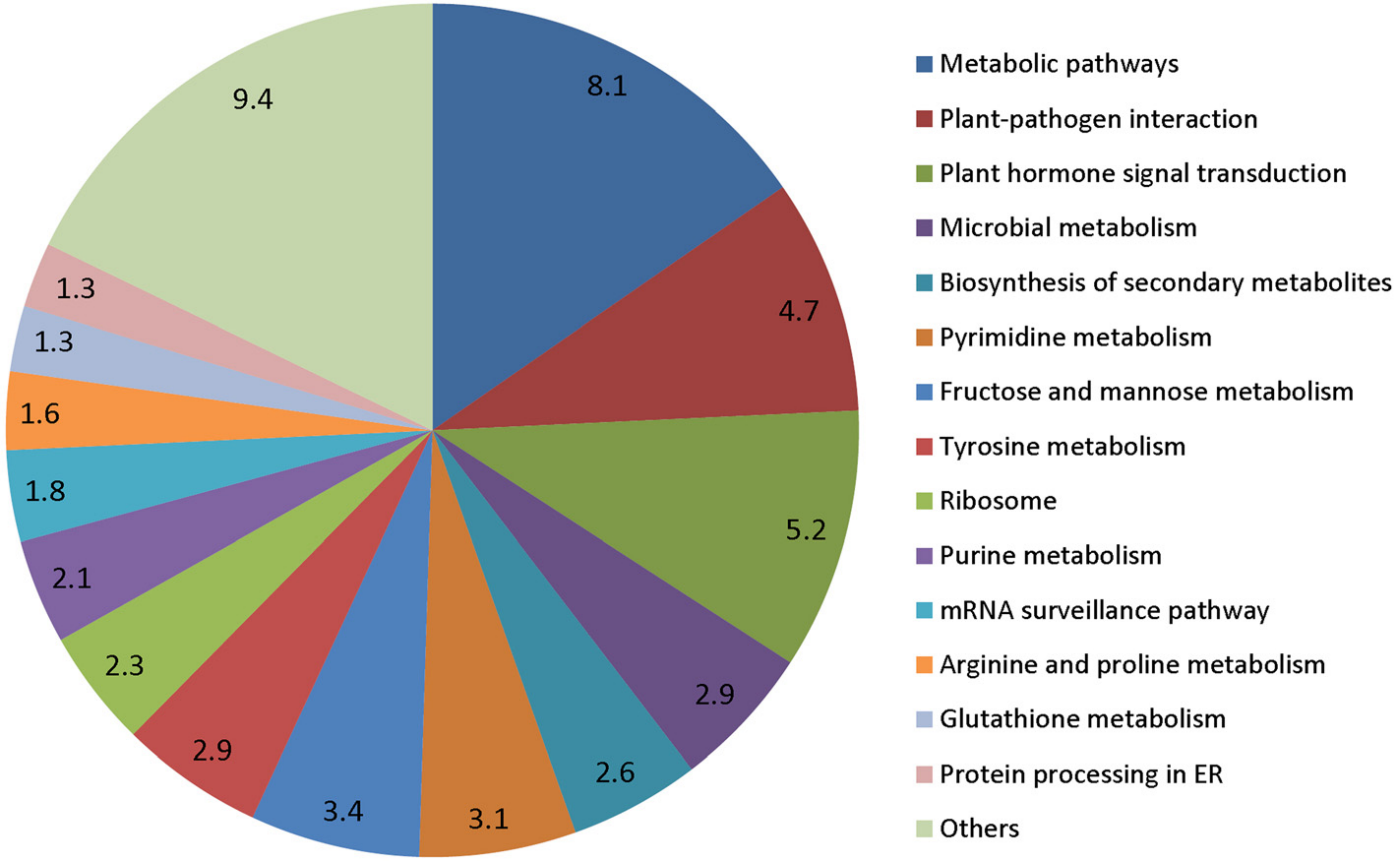
Annotation of miRNA targets based on KEGG orthology. Values are the percentage of target genes in different functional categories

To further evaluate the completeness of the miRNA transcriptome and benefits of the annotation of the target candidates of known and novel miRNAs, all annotated sequences from poplar were identified by KEGG pathway groups. A total of 629 candidates from multiple KO pathways were identified according to *P*-value and *Q*-value from the KEGG database (Fig. 9; Additional file 8: Table S7). We were able to enrich 41 miRNA families together targeting those candidate genes in 107 major pathways related to metabolism of starch synthesis, amino acid synthesis, protein synthesis, plant-pathogen interaction, and hormone signal transduction, etc. In addition, biosynthesis of secondary metabolites, and fructose and mannose metabolism pathways which are important for fruit maturity also existed. To support the participation of KEGG pathways in pomegranate fruit development, the previous evidence shows that edible part of the pomegranate arils contain 10 % total sugars comprising fructose and glucose, ascorbic acid, citric acid, bioactive compounds such as phenolics and flavonoids, principally anthocyanins [56]. Specifically, novel miRNAs were found to be involved in different steps of multiple pathways (Additional file 9: Figure S1), including ascorbate metabolism (conversion of L-ascorbate to L-dehydroascorbate), fatty acid metabolism, carbon fixation (change of ribose 5-phosphate to ribulose 5-phosphate), and RNA transport (by regulating members involved in nuclear pore complex and exon junction complex). More importantly, novel miRNA members participated in plant hormone transduction pathways such as auxin (regulating genes *TIR1*, *ARF* and *SAUR*), cytokinin (*CRE1* and *A-ARR*), gibberellin (*DELLA*), abscisic acid (*PP2C*), brassinosteroid (*BAK1/BRI1* and *BZR1/2*), and jasmonic acid (*JAZ* and *MYC2*). These key hormone related pathways may participate in synthesis of various phytocompounds in mature pomegranate fruit as the gene ontology suggested. KEGG pathway analysis showed 14 candidates in fructose and mannose metabolism, 1 in carbon fixation, 23 members in biosynthesis of secondary metabolites, and 4 candidates for the sucrose and starch metabolism. Altogether, the pomegranate fruit quality is largely impacted by the composition of sugar and acid, which is one of the most significant fruit development characteristics.

## Conclusions

We used small RNA-sequencing of pomegranate with Illumina Hiseq2000 sequencing and identified 10 novel miRNAs. We reveal the differential expression of a few predominately expressed miRNAs and their variants in different developmental stages of fruit. This is the first report to investigate sRNAs in pomegranate, with a large number found as known and novel miRNAs. By searching the poplar genome, 288 putative target genes were predicted for the 10 novel miRNAs and then annotated by using GO and KEGG databases to explore their putative functions in different metabolic pathways. We revealed several fruit development pathways including sugar and acid, and plant hormone signaling. This identification of novel miRNAs in pomegranate will be valuable for further understanding the functions and regulatory mechanisms of miRNAs in other related plant species.

## Additional files

Additional file 1: Table S8. Primers used in this study for stem-loop RT-qPCR. (XLSX 10 kb) Additional file 2: Table S1. Length distribution and frequency of small RNA tags. (XLSX 10 kb) Additional file 3: Table S2. Total counts of variants of known miRNAs. (XLSX 11 kb) Additional file 4: Table S3. List of novel miRNAs, their precursors with hairpin structure sequences. (XLSX 10 kb) Additional file 5: Table S4. Novel miRNAs with the first nucleotide bias for 18- to 26-nt small RNAs. (XLSX 8 kb) Additional file 6: Table S5. miRNA nucleotide bias at each position of 24-nt small RNAs. (XLSX 9 kb) Additional file 7: Table S6. List of target candidates of novel miRNAs. (XLSX 112 kb) Additional file 8: Table S7. List of different KEGG pathways. (XLSX 19 kb) Additional file 9: Figure S1. List of pathways with participation of miRNAs. (PDF 343 kb)
