# Supplementary material for: Genome-wide identification of microRNAs in pomegranate (Punica granatum L.) by high-throughput sequencing
Source: BMC Plant Biol. 2016 May 26;16:122. doi: 10.1186/s12870-016-0807-3 (PMC4880961; doi:10.1186/s12870-016-0807-3)
Supplement: Additional file 9: Figure S1. — List of pathways with participation of miRNAs. (PDF 343 kb) [file 12870_2016_807_MOESM9_ESM.pdf]

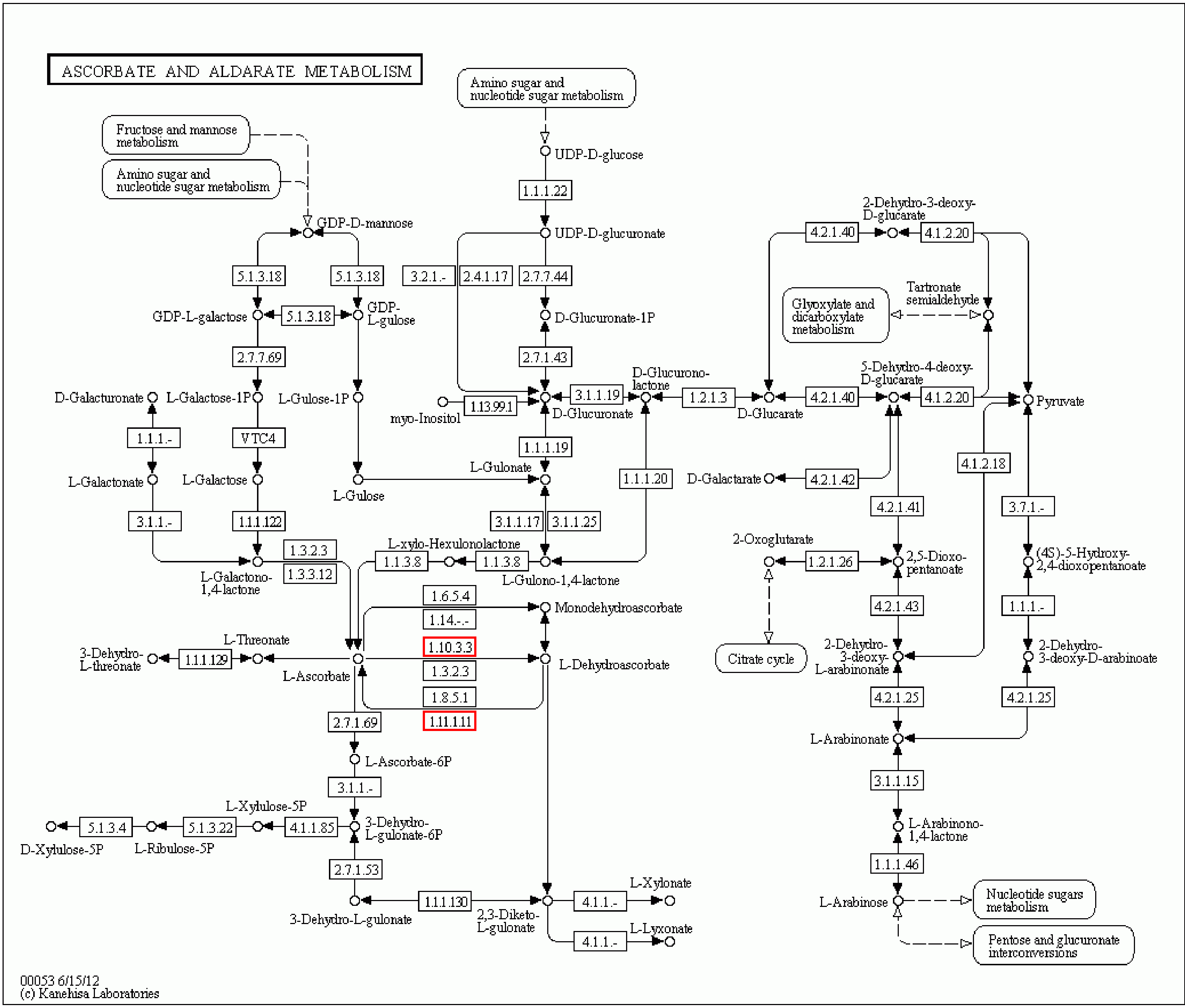
ASCORBATE AND ALDARATE METABOLISM
Amino sugar and nucleotide sugar metabolism
Fructose and mannose metabolism
Amino sugar and nucleotide sugar metabolism
UDP-D-glucose
1.1.1.22
GDP-D-mannose
UDP-D-glucuronate
5.1.3.18
5.1.3.18
3.2.1.-
2.4.1.17
2.7.7.44
GDP-L-galactose
5.1.3.18
GDP-L-gulose
D-Glucuronate-1P
2.7.7.69
2.7.1.43
D-Galacturonate
L-Galactose-1P
L-Gulose-1P
myo-Inositol
1.13.99.1
D-Glucuronate
3.1.1.19
D-Glucurono-lactone
1.2.1.3
D-Glucarate
4.2.1.40
5-Dehydro-4-deoxy-D-glucarate
4.1.2.20
Pyruvate
2-Dehydro-3-deoxy-D-glucarate
4.2.1.40
4.1.2.20
Glyoxylate and dicarboxylate metabolism
Tartronate semialdehyde
1.1.1.-
VTC4
1.1.1.19
L-Galactonate
L-Galactose
L-Gulonate
L-Gulose
1.1.1.20
D-Galactarate
4.2.1.42
4.1.2.18
4.2.1.41
3.7.1.-
3.1.1.-
1.1.1.122
3.1.1.17
3.1.1.25
1.3.2.3
1.3.3.12
L-xylo-Hexulonolactone
1.1.3.8
1.1.3.8
L-Galactono-1,4-lactone
L-Gulono-1,4-lactone
2-Oxoglutarate
1.2.1.26
2,5-Dioxo-pentanoate
(4S)-5-Hydroxy-2,4-dioxopentanoate
1.6.5.4
1.14.-.-
Monodehydroascorbate
4.2.1.43
1.1.1.-
L-Threonate
3-Dehydro-L-threonate
1.1.1.129
L-Ascorbate
1.10.3.3
1.3.2.3
1.8.5.1
1.11.1.11
L-Dehydroascorbate
Citrate cycle
2-Dehydro-3-deoxy-L-arabinonate
2-Dehydro-3-deoxy-D-arabinonate
4.2.1.25
4.2.1.25
2.7.1.69
L-Ascorbate-6P
L-Arabinonate
3.1.1.-
3.1.1.15
L-Xylulose-5P
5.1.3.4
5.1.3.22
4.1.1.85
3-Dehydro-L-gulonate-6P
D-Xylulose-5P
L-Ribulose-5P
L-Arabinono-1,4-lactone
2.7.1.53
1.1.1.46
3-Dehydro-L-gulonate
1.1.1.130
2,3-Diketo-L-gulonate
4.1.1.-
L-Xylonate
4.1.1.-
L-Lyxonate
L-Arabinose
Nucleotide sugars metabolism
Pentose and glucuronate interconversions
00053 6/15/12
(c) Kanehisa Laboratories

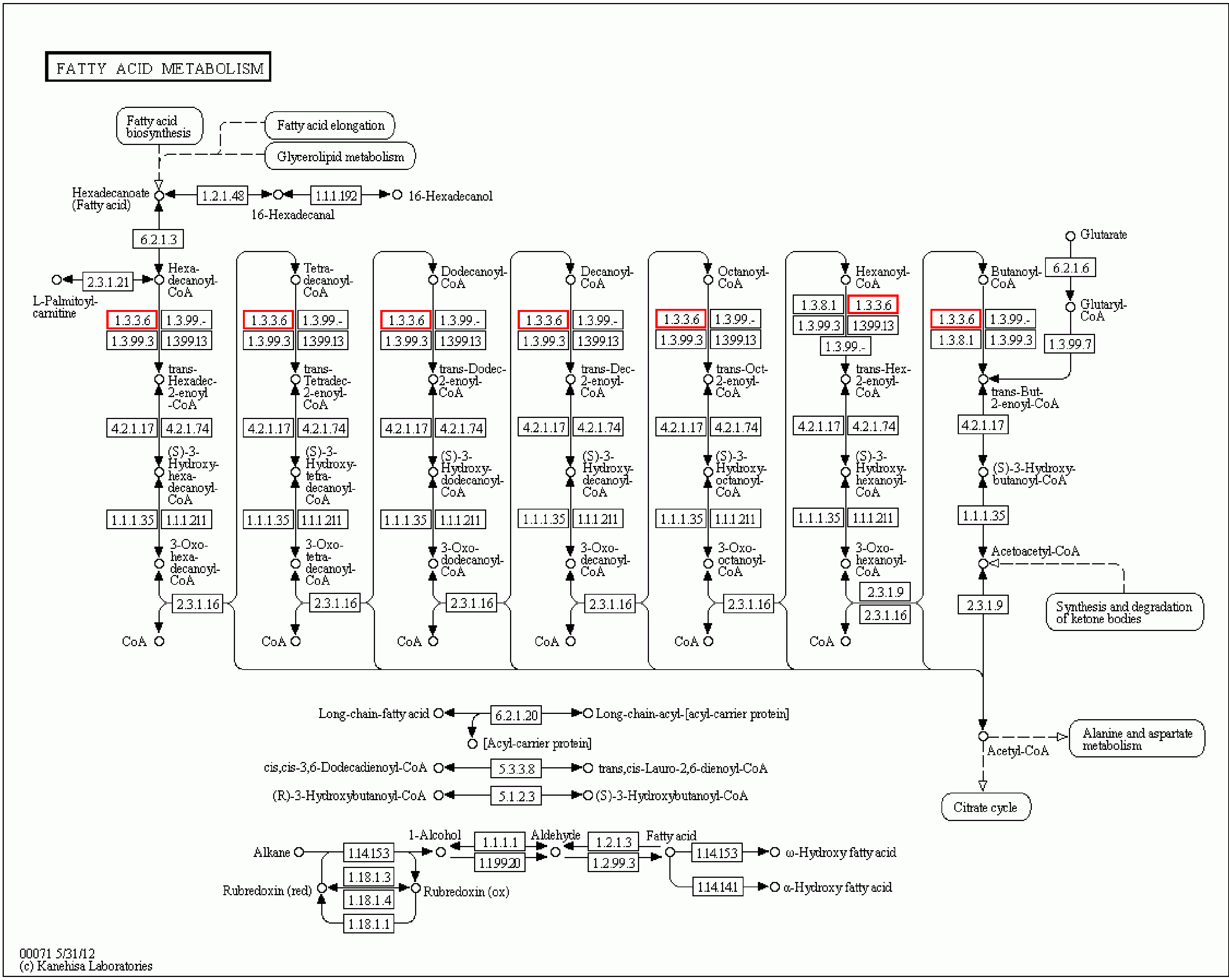
FATTY ACID METABOLISM
Fatty acid biosynthesis
Fatty acid elongation
Glycerolipid metabolism
Hexadecanoate (Fatty acid)
1.2.1.48
1.1.1.192
16-Hexadecanol
16-Hexadecanal
6.2.1.3
2.3.1.21
L-Palmitoyl-carnitine
Hexa-decanoyl-CoA
Tetra-decanoyl-CoA
Dodecanoyl-CoA
Decanoyl-CoA
Octanoyl-CoA
Hexanoyl-CoA
Butanoyl-CoA
Glutarate
6.2.1.6
Glutaryl-CoA
1.3.99.7
1.3.3.6
1.3.99.-
1.3.99.3
1.3.99.13
1.3.8.1
trans-Hexadec-2-enoyl-CoA
trans-Tetradec-2-enoyl-CoA
trans-Dodec-2-enoyl-CoA
trans-Dec-2-enoyl-CoA
trans-Oct-2-enoyl-CoA
trans-Hex-2-enoyl-CoA
trans-But-2-enoyl-CoA
4.2.1.17
4.2.1.74
(S)-3-Hydroxy-hexa-decanoyl-CoA
(S)-3-Hydroxy-tetra-decanoyl-CoA
(S)-3-Hydroxy-dodecanoyl-CoA
(S)-3-Hydroxy-decanoyl-CoA
(S)-3-Hydroxy-octanoyl-CoA
(S)-3-Hydroxy-hexanoyl-CoA
(S)-3-Hydroxy-butanoyl-CoA
1.1.1.35
1.1.1.211
3-Oxo-hexa-decanoyl-CoA
3-Oxo-tetra-decanoyl-CoA
3-Oxo-dodecanoyl-CoA
3-Oxo-decanoyl-CoA
3-Oxo-octanoyl-CoA
3-Oxo-hexanoyl-CoA
Acetoacetyl-CoA
2.3.1.16
2.3.1.9
CoA
Synthesis and degradation of ketone bodies
Long-chain-fatty acid
6.2.1.20
Long-chain-acyl-[acyl-carrier protein]
[Acyl-carrier protein]
Acetyl-CoA
Alanine and aspartate metabolism
Citrate cycle
cis,cis-3,6-Dodecadienoyl-CoA
5.3.3.8
trans,cis-Lauro-2,6-dienoyl-CoA
(R)-3-Hydroxybutanoyl-CoA
5.1.2.3
(S)-3-Hydroxybutanoyl-CoA
Alkane
1.14.15.3
1-Alcohol
1.1.1.1
1.1.99.20
Aldehyde
1.2.1.3
1.2.99.3
Fatty acid
ω-Hydroxy fatty acid
1.14.14.1
α-Hydroxy fatty acid
Rubredoxin (red)
1.18.1.3
1.18.1.4
1.18.1.1
Rubredoxin (ox)
00071 5/31/12
(c) Kanehisa Laboratories

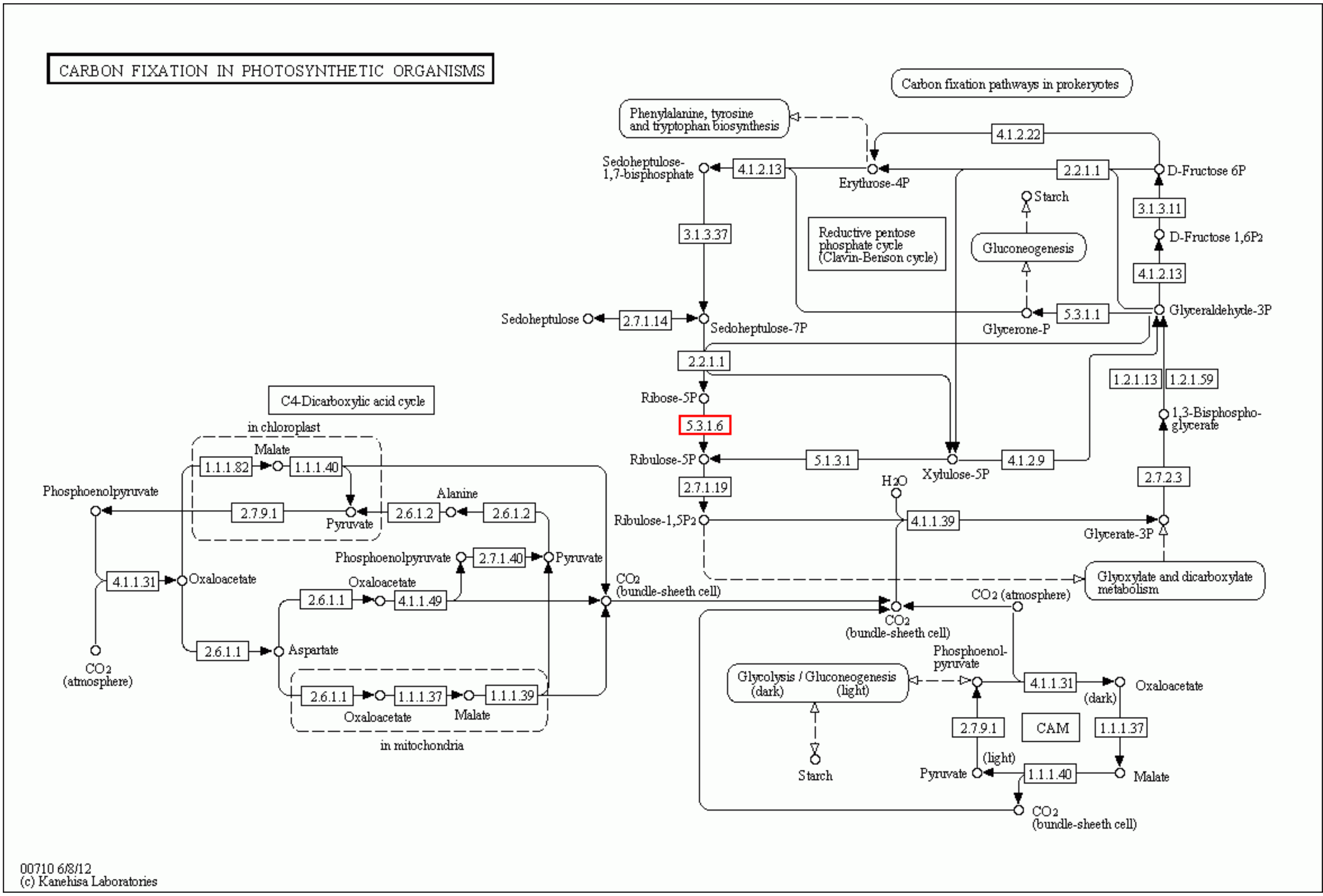
CARBON FIXATION IN PHOTOSYNTHETIC ORGANISMS
Carbon fixation pathways in prokaryotes
Phenylalanine, tyrosine and tryptophan biosynthesis
4.1.2.22
Sedoheptulose-1,7-bisphosphate
4.1.2.13
Erythrose-4P
2.2.1.1
D-Fructose 6P
Starch
3.1.3.11
3.1.3.37
Reductive pentose phosphate cycle (Clavin-Benson cycle)
Gluconeogenesis
D-Fructose 1,6P2
4.1.2.13
Sedoheptulose
2.7.1.14
Sedoheptulose-7P
Glycerone-P
5.3.1.1
Glyceraldehyde-3P
2.2.1.1
1.2.1.13
1.2.1.59
C4-Dicarboxylic acid cycle
Ribose-5P
1,3-Bisphospho-glycerate
in chloroplast
5.3.1.6
Malate
Ribulose-5P
5.1.3.1
Xylulose-5P
4.1.2.9
2.7.2.3
1.1.1.82
1.1.1.40
Phosphoenolpyruvate
Alanine
2.7.1.19
H2O
2.7.9.1
2.6.1.2
2.6.1.2
Ribulose-1,5P2
4.1.1.39
Pyruvate
Glycerate-3P
Phosphoenolpyruvate
2.7.1.40
Pyruvate
4.1.1.31
Oxaloacetate
Oxaloacetate
CO2 (bundle-sheath cell)
Glyoxylate and dicarboxylate metabolism
2.6.1.1
4.1.1.49
CO2 (atmosphere)
CO2 (bundle-sheath cell)
2.6.1.1
Aspartate
Phosphoenol-pyruvate
CO2 (atmosphere)
2.6.1.1
1.1.1.37
1.1.1.39
Glycolysis / Gluconeogenesis (dark) (light)
4.1.1.31
Oxaloacetate
Oxaloacetate
Malate
(dark)
in mitochondria
2.7.9.1
CAM
1.1.1.37
Starch
(light)
Pyruvate
1.1.1.40
Malate
CO2 (bundle-sheeth cell)
00710 6/8/12
(c) Kanehisa Laboratories

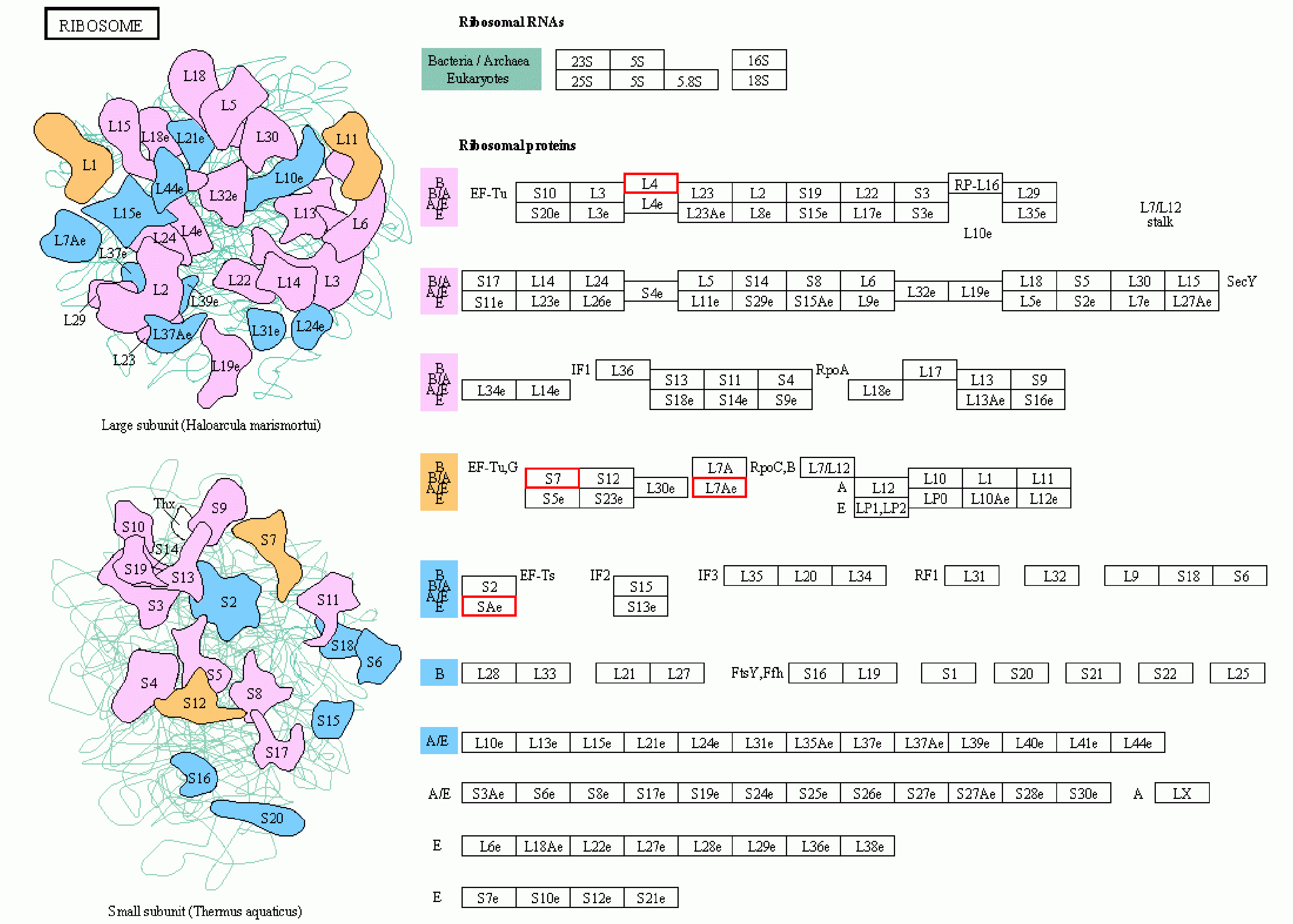


03010 11/29/10

(c) Kanehisa Laboratories

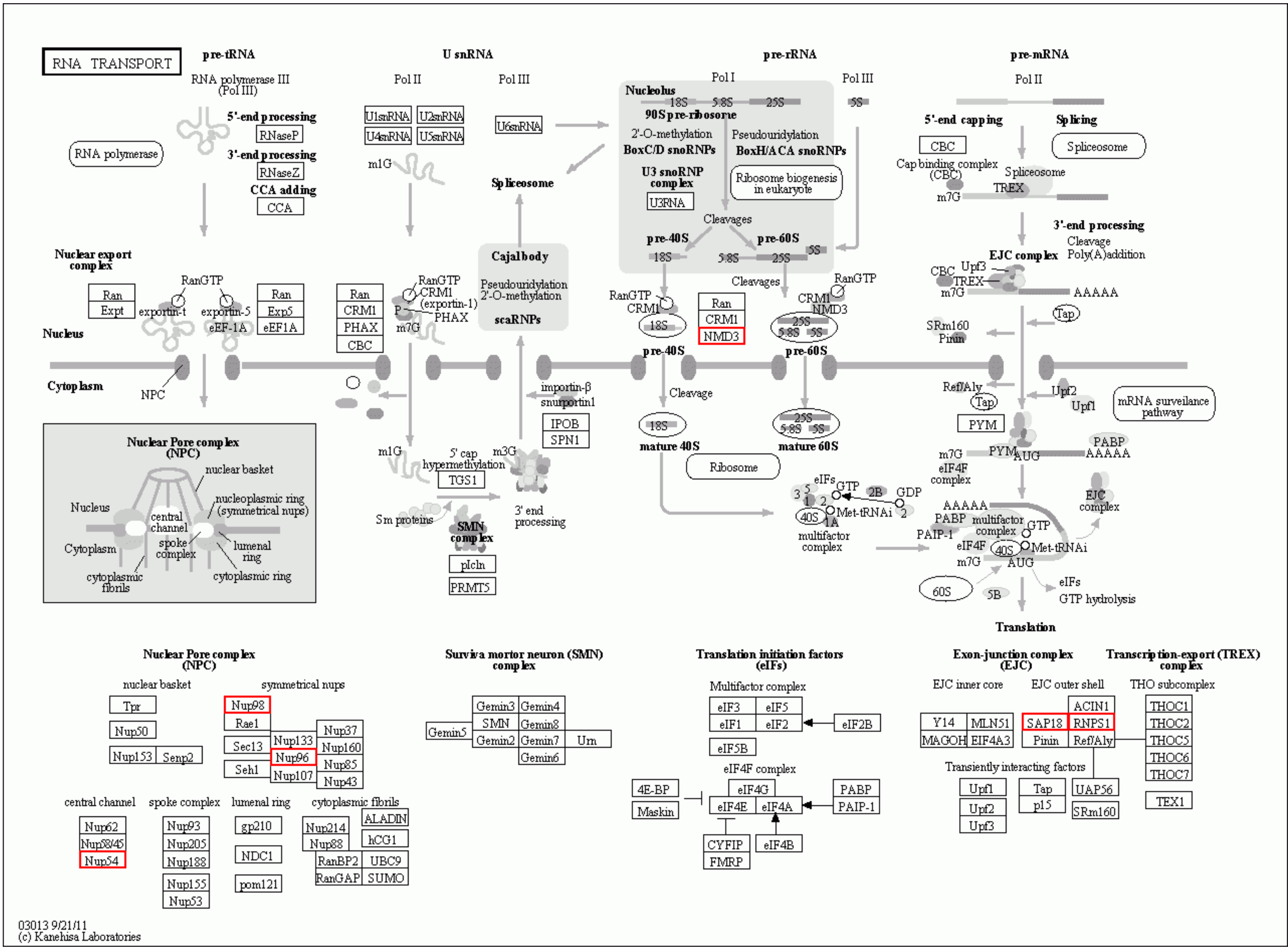
RNA TRANSPORT
pre-tRNA
RNA polymerase III (Pol III)
5'-end processing
RNaseP
3'-end processing
RNaseZ
CCA adding
CCA
RNA polymerase
U snRNA
Pol II
Pol III
U1snRNA
U2snRNA
U4snRNA
U5snRNA
U6snRNA
m1G
Spliceosome
pre-rRNA
Pol I
Pol III
Nucleolus
18S
5.8S
25S
5S
90S pre-ribosome
2'-O-methylation
BoxC/D snoRNPs
Pseudouridylation
BoxH/ACA snoRNPs
U3 snoRNP complex
U3RNA
Ribosome biogenesis in eukaryote
Cleavages
pre-40S
pre-60S
pre-mRNA
5'-end capping
CBC
Cap binding complex (CBC)
Splicing
Spliceosome
TREX
m7G
3'-end processing
Cleavage
Poly(A)addition
EJC complex
Upf3
AAAAA
Tap
SRm160
Pinin
Nuclear export complex
RanGTP
Ran
Expt
exportin-t
exportin-5
Exp5
eEF-1A
eEF1A
CRM1
PHAX
CBC
(exportin-1)
P
Cajal body
Pseudouridylation
2'-O-methylation
scaRNPs
NMD3
Nucleus
Cytoplasm
NPC
importin-β
snurportin1
IPOB
SPN1
Cleavage
mature 40S
mature 60S
Ribosome
Ref/Aly
Upf2
Upf1
PYM
mRNA surveilance pathway
PABP
AUG
eIF4F complex
5' cap hypermethylation
m3G
TGS1
Sm proteins
SMN complex
pICln
PRMT5
3' end processing
eIFs
GTP
2B
GDP
Met-tRNAi
40S
1A
multifactor complex
PAIP-1
eIF4F
EJC complex
60S
5B
GTP hydrolysis
Translation
Nuclear Pore complex (NPC)
nuclear basket
nucleoplasmic ring (symmetrical nups)
central channel
spoke complex
lumenal ring
cytoplasmic fibrils
cytoplasmic ring
Nuclear Pore complex (NPC)
nuclear basket
symmetrical nups
Tpr
Nup50
Nup153
Senp2
Nup98
Rae1
Sec13
Seh1
Nup133
Nup96
Nup107
Nup37
Nup160
Nup85
Nup43
central channel
spoke complex
lumenal ring
cytoplasmic fibrils
Nup62
Nup58/45
Nup54
Nup93
Nup205
Nup188
Nup155
Nup53
gp210
NDC1
pom121
Nup214
Nup88
ALADIN
hCG1
RanBP2
UBC9
RanGAP
SUMO
Surviva mortor neuron (SMN) complex
Gemin3
Gemin4
SMN
Gemin8
Gemin5
Gemin2
Gemin7
Urn
Gemin6
Translation initiation factors (eIFs)
Multifactor complex
eIF3
eIF5
eIF1
eIF2
eIF2B
eIF5B
eIF4F complex
4E-BP
Maskin
eIF4G
eIF4E
eIF4A
PABP
PAIP-1
CYFIP
FMRP
eIF4B
Exon-junction complex (EJC)
EJC inner core
EJC outer shell
Y14
MLN51
MAGOH
EIF4A3
ACIN1
SAP18
RNPS1
Pinin
Ref/Aly
Transiently interacting factors
Upf1
Upf2
Upf3
Tap
p15
UAP56
SRm160
Transcription-export (TREX) complex
THO subcomplex
THOC1
THOC2
THOC5
THOC6
THOC7
TEX1
03013 9/21/11
(c) Kanehisa Laboratories

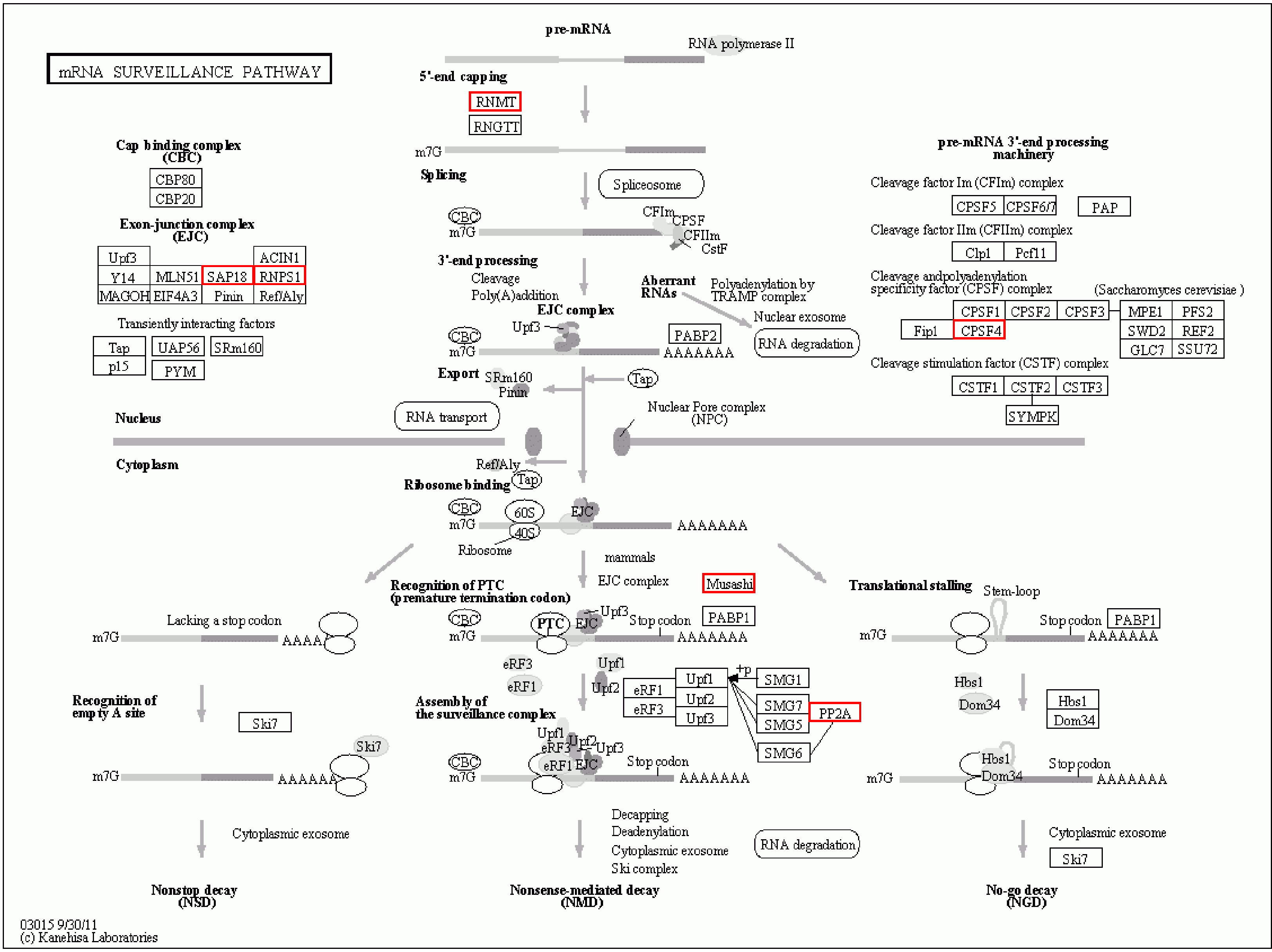
mRNA SURVEILLANCE PATHWAY
pre-mRNA
RNA polymerase II
5'-end capping
RNMT
RNGTT
m7G
Splicing
Spliceosome
Cap binding complex (CBC)
CBP80
CBP20
Exon-junction complex (EJC)
Upf3
ACIN1
Y14
MLN51
SAP18
RNPS1
MAGOH
EIF4A3
Pinin
Ref/Aly
Transiently interacting factors
Tap
p15
UAP56
SRm160
PYM
CBC
CFIm
CPSF
CFIIm
CstF
3'-end processing
Cleavage
Poly(A)addition
EJC complex
Upf3
PABP2
AAAAAAA
Aberrant RNAs
Polyadenylation by TRAMP complex
Nuclear exosome
RNA degradation
pre-mRNA 3'-end processing machinery
Cleavage factor Im (CFIm) complex
CPSF5
CPSF6/7
PAP
Cleavage factor IIm (CFIIm) complex
Clp1
Pcf11
Cleavage and polyadenylation specificity factor (CPSF) complex
(Saccharomyces cerevisiae)
CPSF1
CPSF2
CPSF3
MPE1
PFS2
Fip1
CPSF4
SWD2
REF2
GLC7
SSU72
Cleavage stimulation factor (CSTF) complex
CSTF1
CSTF2
CSTF3
SYMPK
Export
SRm160
Pinin
Tap
RNA transport
Nuclear Pore complex (NPC)
Nucleus
Cytoplasm
Ref/Aly
Tap
Ribosome binding
60S
40S
EJC
Ribosome
mammals
EJC complex
Musashi
Recognition of PTC (premature termination codon)
Translational stalling
Stem-loop
Lacking a stop codon
AAAA
PTC
Stop codon
PABP1
eRF3
eRF1
Upf1
Upf2
eRF1
eRF3
Upf1
Upf2
Upf3
+p
SMG1
SMG7
SMG5
SMG6
PP2A
Assembly of the surveillance complex
Recognition of empty A site
Ski7
AAAAAA
Hbs1
Dom34
Cytoplasmic exosome
Decapping
Deadenylation
Cytoplasmic exosome
Ski complex
RNA degradation
Nonstop decay (NSD)
Nonsense-mediated decay (NMD)
No-go decay (NGD)
03015 9/30/11
(c) Kanehisa Laboratories

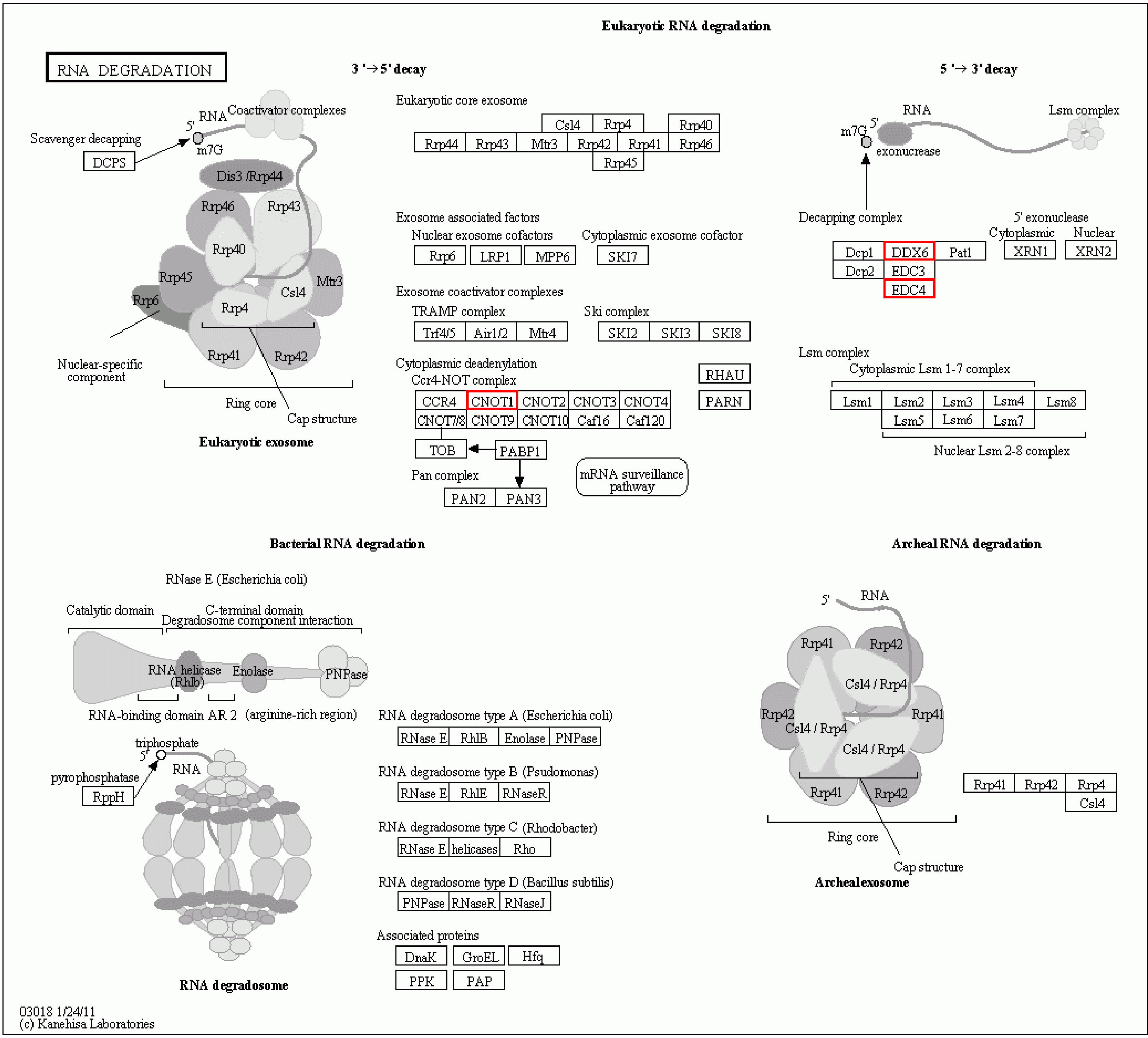
Eukaryotic RNA degradation
RNA DEGRADATION
3 '→ 5' decay
5 '→ 3' decay
Scavenger decapping
DCPS
RNA
Coactivator complexes
m7G
Dis3 /Rrp44
Rrp46
Rrp43
Rrp40
Rrp45
Rrp6
Mtr3
Csl4
Rrp4
Rrp41
Rrp42
Nuclear-specific component
Ring core
Cap structure
Eukaryotic exosome
Eukaryotic core exosome
Csl4
Rrp4
Rrp40
Rrp44
Rrp43
Mtr3
Rrp42
Rrp41
Rrp46
Rrp45
Exosome associated factors
Nuclear exosome cofactors
Cytoplasmic exosome cofactor
Rrp6
LRP1
MPP6
SKI7
Exosome coactivator complexes
TRAMP complex
Ski complex
Trf4/5
Air1/2
Mtr4
SKI2
SKI3
SKI8
Cytoplasmic deadenylation
Ccr4-NOT complex
RHAU
CCR4
CNOT1
CNOT2
CNOT3
CNOT4
PARN
CNOT7/8
CNOT9
CNOT10
Caf16
Caf120
TOB
PABP1
Pan complex
mRNA surveillance pathway
PAN2
PAN3
RNA
Lsm complex
m7G
exonucrease
Decapping complex
5' exonuclease
Cytoplasmic
Nuclear
Dcp1
DDX6
Pat1
XRN1
XRN2
Dcp2
EDC3
EDC4
Lsm complex
Cytoplasmic Lsm 1-7 complex
Lsm1
Lsm2
Lsm3
Lsm4
Lsm8
Lsm5
Lsm6
Lsm7
Nuclear Lsm 2-8 complex
Bacterial RNA degradation
RNase E (Escherichia coli)
Catalytic domain
C-terminal domain
Degradosome component interaction
RNA helicase (Rhlb)
Enolase
PNPase
RNA-binding domain AR 2
(arginine-rich region)
triphosphate
pyrophosphatase
RppH
RNA
RNA degradosome
RNA degradosome type A (Escherichia coli)
RNase E
RhlB
Enolase
PNPase
RNA degradosome type B (Psudomonas)
RNase E
RhlE
RNaseR
RNA degradosome type C (Rhodobacter)
RNase E
helicases
Rho
RNA degradosome type D (Bacillus subtilis)
PNPase
RNaseR
RNaseJ
Associated proteins
DnaK
GroEL
Hfq
PPK
PAP
Archeal RNA degradation
RNA
Rrp41
Rrp42
Csl4 / Rrp4
Rrp42
Rrp41
Csl4 / Rrp4
Csl4 / Rrp4
Rrp41
Rrp42
Ring core
Cap structure
Archealexosome
Rrp41
Rrp42
Rrp4
Csl4
03018 1/24/11
(c) Kanehisa Laboratories

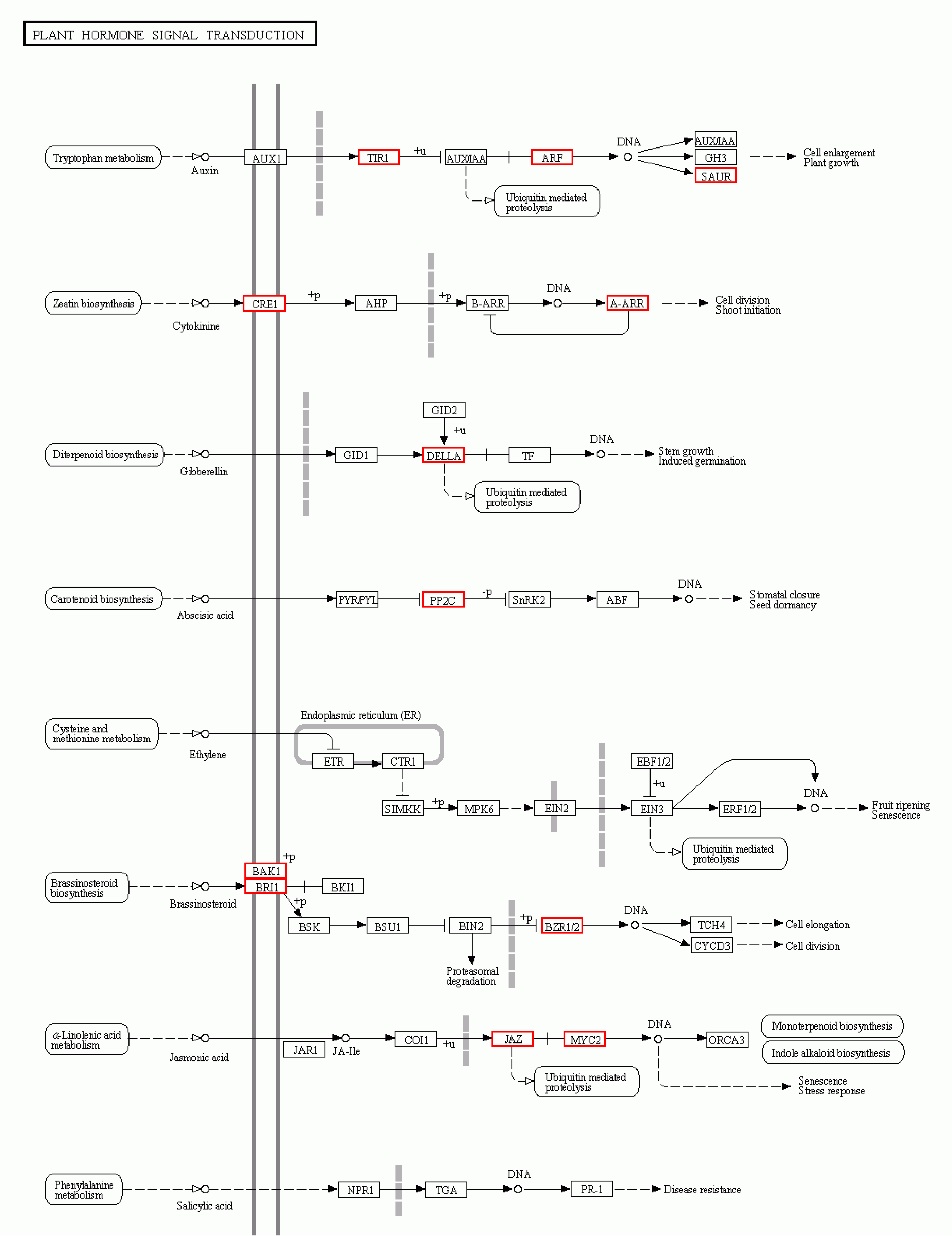
PLANT HORMONE SIGNAL TRANSDUCTION
Tryptophan metabolism
Auxin
AUX1
TIR1
+u
AUX/IAA
ARF
DNA
AUX/IAA
GH3
SAUR
Cell enlargement
Plant growth
Ubiquitin mediated proteolysis
Zeatin biosynthesis
Cytokinine
CRE1
+p
AHP
+p
B-ARR
DNA
A-ARR
Cell division
Shoot initiation
GID2
+u
Diterpenoid biosynthesis
Gibberellin
GID1
DELLA
TF
DNA
Stem growth
Induced germination
Ubiquitin mediated proteolysis
Carotenoid biosynthesis
Abscisic acid
PYR/PYL
PP2C
-p
SnRK2
ABF
DNA
Stomatal closure
Seed dormancy
Endoplasmic reticulum (ER)
Cysteine and methionine metabolism
Ethylene
ETR
CTR1
SIMKK
+p
MPK6
EIN2
EBF1/2
+u
EIN3
ERF1/2
DNA
Fruit ripening
Senescence
Ubiquitin mediated proteolysis
+p
BAK1
Brassinosteroid biosynthesis
Brassinosteroid
BRI1
BKI1
+p
BSK
BSU1
BIN2
+p
BZR1/2
DNA
TCH4
Cell elongation
CYCD3
Cell division
Proteasomal degradation
α-Linolenic acid metabolism
Jasmonic acid
JAR1
JA-Ile
COI1
+u
JAZ
MYC2
DNA
ORCA3
Monoterpenoid biosynthesis
Indole alkaloid biosynthesis
Ubiquitin mediated proteolysis
Senescence
Stress response
Phenylalanine metabolism
Salicylic acid
NPR1
TGA
DNA
PR-1
Disease resistance
